# Soil domestication by rice cultivation results in plant-soil feedback through shifts in soil microbiota

**DOI:** 10.1186/s13059-019-1825-x

**Published:** 2019-10-24

**Authors:** Joseph Edwards, Christian Santos-Medellín, Bao Nguyen, John Kilmer, Zachary Liechty, Esteban Veliz, Jiadong Ni, Gregory Phillips, Venkatesan Sundaresan

**Affiliations:** 1 Department of Plant Biology, University of California, Davis, Life Sciences Addition, 1 Shields Ave., Davis, CA 95616 USA; 2 Present Address: Integrative Biology Department, University of Texas, Austin 2415 Speedway, Austin, TX 78712 USA; 3 Department of Agriculture, Arkansas State University, 2105 Aggie Rd., Jonesboro, AR 72401 USA; 4 Department of Plant Sciences, University of California, Davis, 1 Shields Ave., Davis, CA 95616 USA

## Abstract

**Background:**

Soils are a key component of agricultural productivity, and soil microbiota determine the availability of many essential plant nutrients. Agricultural domestication of soils, that is, the conversion of previously uncultivated soils to a cultivated state, is frequently accompanied by intensive monoculture, especially in the developing world. However, there is limited understanding of how continuous cultivation alters the structure of prokaryotic soil microbiota after soil domestication, including to what extent crop plants impact soil microbiota composition, and how changes in microbiota composition arising from cultivation affect crop performance.

**Results:**

We show here that continuous monoculture (> 8 growing seasons) of the major food crop rice under flooded conditions is associated with a pronounced shift in soil bacterial and archaeal microbiota structure towards a more consistent composition, thereby domesticating microbiota of previously uncultivated sites. Aside from the potential effects of agricultural cultivation practices, we provide evidence that rice plants themselves are important drivers of the domestication process, acting through selective enrichment of specific taxa, including methanogenic archaea, in their rhizosphere that differ from those of native plants growing in the same environment. Furthermore, we find that microbiota from soils domesticated by rice cultivation contribute to plant-soil feedback, by imparting a negative effect on rice seedling vigor.

**Conclusions:**

Soil domestication through continuous monoculture cultivation of rice results in compositional changes in the soil microbiota, which are in part driven by the rice plants. The consequences include a negative impact on plant performance and increases in greenhouse gas emitting microbes.

## Introduction

Plant roots are colonized by complex microbiota that are largely derived from the surrounding soil [1–4]. Root-associated microbiota can benefit the host plant by improving nutrient availability [5], excluding or defending against pathogens [6], and promoting growth by influencing plant hormone pathways [7]. Root-associated microbiota can also confer adverse effects to plant growth. Studies using soils and plants from natural ecosystems have found that plants grown in conspecific soil, that is, soil in which a specific plant species was previously grown, can exhibit reduced biomass and productivity compared to plants grown in heterospecific soil [8]. This effect, known as negative plant-soil feedback, is thought to be a product of detrimental microbial colonization [9] as well as a buildup of plant and microbially synthesized toxins [10, 11]. Negative plant-soil feedback has been studied mainly in the context of non-agronomic, terrestrial ecosystems and is thought to be a mechanism which increases biodiversity by limiting exclusion of plants which are less fit than their competitors [12, 13].

Less is known about plant-soil feedback in agricultural settings, particularly in the context of soil domestication, the process of converting an uncultivated soil to a cultivated state, therefore disrupting natural soil ecosystem and geochemical processes [14]. Crop management practices affect root microbial community assemblages [3, 15], and a recent study on a peanut field indicated that crop management, i.e., continuous monoculture vs. rotation, alters soil microbial communities and affects plant physiology [16]. Aerobically grown rice has noticeable yield drop offs over time, a phenomenon known as soil sickness [17, 18]. However, no such phenomenon has been witnessed or reported in flooded rice [18, 19]. A recent study showed that specific maize genotypes can condition cultivated soils by a root exudate component that in turn affects the composition of root-associated microbiota and negatively impacts shoot biomass [20]. In addition, cultivation of maize has been recently reported to restructure soil microbial diversity in prairie soils; however, the observed changes were attributed to agricultural practices rather than driven by maize plants [21]. Arising from these and earlier studies are unresolved but important questions, as to whether detrimental effects originating from altered microbiota are a general feature of intensive agriculture, and to what extent the crop plant itself, as opposed to agricultural practices, drives such changes in the microbiome. Intensive agricultural cultivation will play a pivotal role in meeting the demands of an expanding world population, and it is increasingly more important to understand how soil biotic factors influence crop growth and yield. Yet, we still know very little about how dense, monoculture crop cultivation influences soil microbiota composition and how microbiota patterns may shape variation in crop growth parameters. In this study, we investigated the following three questions: (1) Does long-term rice cultivation change the bacterial and archaeal components of the soil microbiome? (2) Is the rice plant itself a driver of the soil domestication process at the microbial level? (3) What is the impact on host plant vigor of domesticated microbiomes compared to microbiomes of undomesticated soils? The results provide insights into the impacts of continuous cultivation of rice on bacterial and archaeal soil microbiota (from herein referred to as microbiota) and the consequences of soil domestication through agriculture on rice plant vigor.

## Results

### Soil cultivation history impacts plant root microbial assemblages

To evaluate the effect of intensive rice cultivation on the bacterial and archaeal diversity inhabiting the soil-root continuum, we surveyed the prokaryotic taxonomic composition of bulk soil, rhizosphere, and endosphere communities of rice plants grown in cultivated and non-cultivated soils under flooded conditions in a greenhouse (see the “Methods” section). Cultivated soils (from here on referred to as domesticated soils) were harvested from California fields with a history of > 8 seasons of rice monoculture cultivation while uncultivated soils were obtained from two uncultivated sites adjacent to rice fields (locations for each site are plotted in Additional file 1: Figure S1A). Soil chemistry profiles from each domesticated and uncultivated soil revealed that geography, rather than soil history, largely determined soil chemical properties (Additional file 1: Figure S1B,C, Additional file 2: Table S1). Each uncultivated site supported differing sets of native plant species (Additional file 1: Figure S1D) with minimal overlap.

**Fig. 1:**
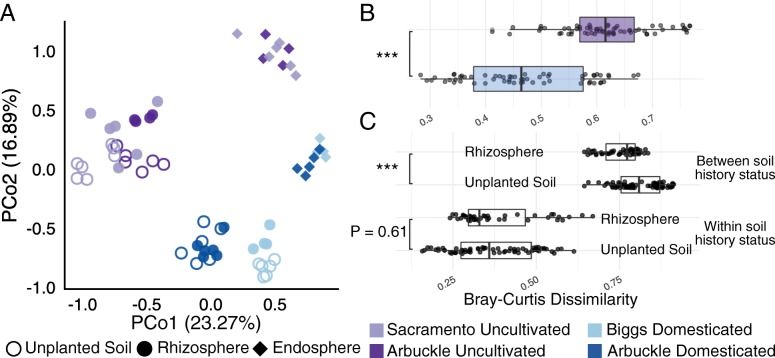
Root microbiota assembly in rice plants domesticates uncultivated soil communities. **a** Principal coordinate analysis of bulk soil, rhizosphere, and endosphere communities of rice plants grown in uncultivated (purple points) and domesticated (blue points) soils. Beta-diversity patterns are based on Bray-Curtis dissimilarities. **b** Distribution of pairwise BC dissimilarities between bulk soil and rhizosphere communities across soil histories. **c** Distribution of pairwise BC dissimilarities between (upper panel) and within (bottom panel) soil history status in the rhizosphere and bulk soil communities. In both **b** and **c**, asterisks indicate significant differences (one-way ANOVA, ***P < 0.001)

Consistent with our previous results [3, 22, 23], we found a significant root compartment effect on microbial communities (*R*^2^ = 0.22, *P* < 0.001, perMANOVA, Additional file 2: Table S2), when using the Bray-Curtis dissimilarity metric. Root-associated microbiota acquired from uncultivated soils were significantly different and clustered distinctly from those acquired from domesticated soils (Fig. 1a, *R*^2^ = 0.18, *P* < 0.001, perMANOVA, Additional file 2: Table S2). We noticed a significant interaction term between soil history and root compartment (*R*^2^ = 0.05, *P* < 0.001, perMANOVA, Additional file 2: Table S2). Similar patterns were also observed when other dissimilarity metrics were calculated (Additional file 1: Figure S2, Additional file 2: Table S3). Although prokaryotic microbiota within each compartment were significantly affected by soil cultivation history, the rhizosphere communities were more affected by soil history compared to endosphere communities (*R*^2^ = 0.31 vs. 0.27, respectively, *P* = 0.001, perMANOVA, Additional file 2: Table S2). Additionally, we observed significantly more variability in uncultivated bulk soil, rhizosphere, and endosphere microbiota compared to those of domesticated soils (Additional file 1: Figure S3, *P* < 0.05, Tukey’s honest significant difference test on distances to centroid within groups, Additional file 2: Table S4). Because the floristic composition inhabiting a soil may contribute to the soil microbial community composition [24, 25], the variation observed between uncultivated soils could be explained by differences in plant cover between sites (Additional file 1: Figure S1D).

The compositional transition from bulk soil to rhizosphere communities is the first step in root microbiome assembly and involves host-mediated recruitment and depletion of specific soil taxa. To assess if this rhizosphere effect displayed differential trends based on soil domestication status, we compared the pairwise dissimilarities between rhizosphere and bulk soil communities across cultivation histories. Relative to domesticated samples, uncultivated rhizosphere microbiota exhibited significantly greater shifts from their respective bulk soil controls (Fig. 1b, *P* = 7.14 × 10^–26^, ANOVA). This result suggests that, under monoculture cultivation, soil communities are potentially restructured towards a compositional state progressively more similar to the one observed in rhizosphere communities. Comparing across soil history types, we found that rhizosphere prokaryotic microbiota were significantly more similar than those of bulk soil samples (Fig. 1c, “between soil type” panel). We note that this is not an effect of rhizosphere communities displaying less variation than bulk soil communities when comparing within soil history type (Fig. 1c, “within soil type” panel). This pattern indicates that rhizosphere microbiome acquisition reduces the inherent compositional differences between domesticated and uncultivated bulk soil communities and therefore could reflect the initiation of soil domestication in less than one growing season.

We next identified individual taxa responsible for the acquired microbiome differences between plants grown in domesticated and uncultivated soil. We used DESeq2 to identify microbes that were enriched or depleted in the compartments of rice plants grown in domesticated soil vs. uncultivated soil (Additional file 2: Table S5). Because this experiment was carried out in two batches (see the “Methods” section), we modeled each experimental batch separately and found the overlap of OTUs that were significantly enriched in each compartment of domesticated and uncultivated soils between the batches (Additional file 2: Table S6). We found a total of 140 unique OTUs to be enriched in the compartments of plants grown in domesticated soil (95 in the bulk soil, 106 in the rhizosphere, and 16 in the endosphere) while we found 256 OTUs to be enriched in the compartments of rice plants grown in uncultivated soils (163 in the bulk soil, 109 in the rhizosphere, and 83 in the endosphere). Soil cultivation history disproportionately affected the abundance of OTUs from several phyla: OTUs of Euryarchaeota, Armatimonadetes, Acidobacteria, Deltaproteobacteria, Chloroflexi, Firmicutes, and Crenarchaeota were all enriched in the compartments of plants grown in domesticated soils more than expected by chance (Additional file 1: Figure S4, *P* < 0.05, hypergeometric test), while Gamma, Beta, and Alpha Proteobacteria, Gemmatimonadetes, Planctomycetes, and Actinobacteria members were more disproportionately enriched in the microbiomes assembled from uncultivated soils (Additional file 1: Figure S4, adjusted *P* ≤ 0.05, hypergeometric test). Methanogenic archaea were found to be enriched in the compartments of rice plants grown in domesticated soil vs. uncultivated soil. Taken together, these observations support the hypothesis that rice cultivation “domesticates” the microbiome of the soil environment to be more similar to the rice rhizosphere microbiota.

### Rice acquires a distinctive microbiome compared to native plant species

**Fig. 2:**
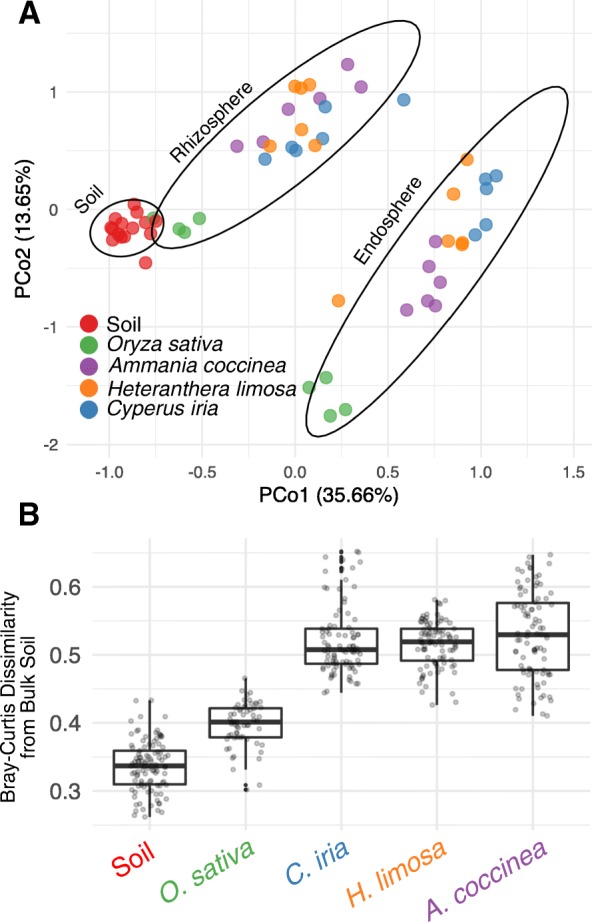
Rice assembles a compositionally distinct root microbiota from native plant species grown in flooded paddy fields. **a** Principal coordinate analysis of soil, rhizosphere, and endosphere communities across rice (*O. sativa*) and three different native plant species: redstem (*A. coccinea*), mudplantain (*H. limosa*), and sedge (*C. iria*). Beta-diversity patterns are based on Bray-Curtis dissimilarities. **b** Bray-Curtis dissimilarity values comparing bulk soil prokaryotic communities to those acquired in the rhizosphere of rice and native plants

Soil domestication in rice fields likely alters the existing soil microbiota through a combination of mechanisms. A legacy of flooding, addition of chemical fertilizers and pesticides, and mechanical disruption by tilling are practices which could influence physiochemical properties of soil and therefore might reshape microbial communities. In addition, host-microbe interactions with the roots of rice, compounded by dense and continuous monoculture, may also play a prominent role in transitioning soil prokaryotic communities from a pre-cultivated to a domesticated status. To address the hypothesis that soil domestication may result at least in part due to host-microbe interactions with rice roots, we compared root-associated microbiomes of rice plants to those of three native plant species growing under the same flooded and managed conditions in a rice field in Jonesboro, Arkansas (see the “Methods” section): *Heteranthera limosa* (blue mud plantain), *Cyperus iria* (flatsedge), and *Ammania coccinea* (valley redstem). These three species are not closely related, with the first two being monocots of the lily and grass families, respectively, and the third a eudicot. A principal coordinate analysis (PCoA) of pairwise Bray-Curtis dissimilarities revealed that samples are distinguishable by root compartment and by plant species (Fig. 2a; compartment: *R*^2^ = 0.42, *P* < 0.001; plant species: *R*^2^ = 0.14, *P* < 0.001, perMANOVA, Additional file 2: Table S7). Similar results were found using alternative dissimilarity metrics (Additional file 1: Figure S5, Additional file 2: Table S8). There was a significant interaction term between compartment and plant species (*R*^2^ = 0.05, *P* < 0.011, Additional file 2: Table S7), suggesting that the magnitude of divergence between microbiota of the different plant species is dependent upon the root compartment. We compared the effect sizes for host species on microbiome composition between each compartment finding that endosphere microbiomes were slightly more affected by host species (*R*^2^ = 0.42, *P* < 0.001, Additional file 2: Table S7) than the rhizosphere microbiome (*R*^2^ = 0.35, *P* < 0.001, Additional file 2: Table S7). In both the rhizosphere and endosphere, rice plants appeared to host microbiota distinct from each native plant, i.e., native plants support microbial communities that are more similar to each other than to rice. We further confirmed that, after excluding rice plants from the analysis, host plant species explained a significant proportion of the observed community variance (rhizosphere: *R*^2^ = 0.23, *P* < 0.001; endosphere: *R*^2^ = 0.28, *P* < 0.001, perMANOVA, Additional file 2: Table S7). These results indicate that rice supports root-associated microbiota distinct from native plants growing in a submerged rice field and that each native plant species is colonized by microbiota divergent from the other surveyed native plant species.

Another observation from the PCoA was that rice rhizosphere samples clustered closer towards the rice field bulk soil samples than did the native plant samples (Fig. 2a). Direct comparisons of rhizosphere community dissimilarities to bulk soil indicated that rice rhizosphere microbiota have greater similarity to bulk soil communities as compared to those of the native plants (Fig. 2b, Additional file 2: Table S9). Taken together, these observations suggest that in addition to flooding and other cultivation practices, rice plants likely have a role in domesticating soil microbiota through selective enrichment and depletion of microbial taxa by roots.

We next investigated which OTUs differentiate the rice microbiome from the native plants by inspecting which microbes have significantly different relative abundances using DESeq2 (Additional file 1: Figure S6A, Additional file 2: Table S10). We determined a core set of rice enriched and depleted microbes through identifying microbes that were commonly enriched or depleted in rice compared to the native plants (solid points in Additional file 1: Figure S6A and three way intersects in Additional file 1: Figure S6B, Additional file 2: Table S11). The set of rice core enriched microbes in the rhizosphere disproportionately belong to Acidobacteria, Chloroflexi, Euryarchaeota, Gemmatimonadetes, Epsilonproteobacteria, and Crenarchaeota (adjusted *P* < 0.05, hypergeometric test; Additional file 1: Figure S7). In the endosphere, the rice core enriched microbes disproportionately belong to Deltaproteobacteria, Firmicutes, Euryarchaeota, Chlorobi, and Spirochaetes (adjusted *P* < 0.05, hypergeometric test; Additional file 1: Figure S7). On the other hand, the core native plant enriched microbes (i.e., microbes consistently depleted from rice roots compared to native plants) disproportionately belonged to Betaproteobacteria, Verrucomicrobia, Bacteroidetes, Planctomycetes, and Gammaproteobacteria in the rhizosphere and Betaproteobacteria and Gammaproteobacteria in the endosphere (adjusted *P* < 0.05, hypergeometric test; Additional file 1: Figure S7).

**Fig. 3:**
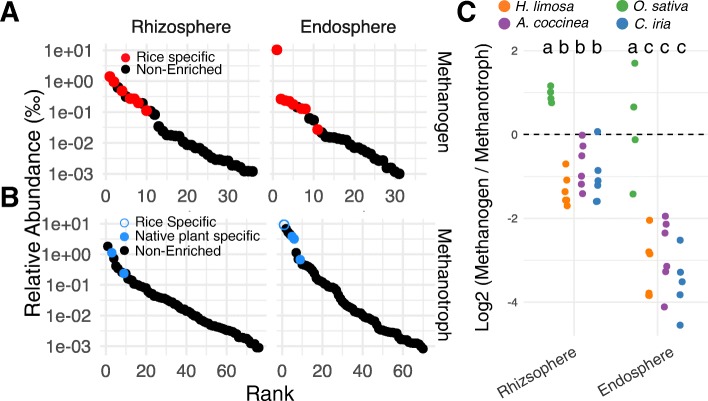
Contrasting enrichment of methanogenic archaea and methanotrophic eubacteria in root-associated communities of rice and native plant species. **a**, **b** Rank abundance curves for methanogens (**a**) and methanotrophs (**b**) in rhizosphere and endosphere communities of rice plants. Colored points represent OTUs differentially abundant between rice and native plants (Wald test, *P* < 0.05 after multiple-comparison adjustment). **c** Methanogen-to-methanotroph log2 ratios in rhizosphere and endosphere communities of rice and native plants. Different letters indicate significant differences between hosts (Tukey test, *P* < 0.05 after multiple-comparison adjustment)

Methanogenic archaea are important contributors to methane emissions from rice paddies. In the set of differentially abundant microbes, we found 7 OTUs belonging to methanogenic taxonomies specifically enriched in the rice rhizosphere and 8 OTUs in the endosphere. Four OTUs were shared between the rhizosphere and endosphere rice core enriched methanogens. Of the 36 methanogenic OTUs detected in the rhizosphere, the rice core enriched OTUs were all within the top 12 most abundant (Fig. 3a). Similarly in the endosphere, of the 31 detectable methanogenic OTUs, the rice core enriched were all within the 11 most abundant (Fig. 3a). We were unable to identify any methanogenic OTUs enriched in the native plants compared to rice.

We next compared methanogenic relative abundance between the root compartments separately for rice plants and the native plants. We found, in general, that the rice rhizosphere hosted a greater relative abundance of methanogens compared to both bulk soil and endosphere communities (Additional file 1: Figure S8), similar to results previously reported by us and others [3, 26]. However, when we performed similar comparisons between the root compartments of the native plants, we found that bulk soils hosted significantly greater abundances of methanogens than rhizosphere and endosphere communities (Additional file 1: Figure S8). Together, these results suggest that rice plants, but not native plants, enrich for methanogenic archaea in the rhizosphere when under flooded conditions.

Methanotrophic eubacteria use methane as an energy source, thus counteracting methane emissions. We found no rice-specific methanotrophic OTUs in the rhizosphere dataset, and only one methanotrophic OTU in the rice core enriched endosphere microbiota, although this particular OTU was the most abundant methanotrophic bacteria in our endosphere dataset (Fig. 3b). The core native plant enriched microbes contained more methanotrophs: in the rhizosphere set, we found 2 methanotrophic OTUs while we found 3 in the endosphere set. The core native plant methanotrophs were among the most abundant methanotrophs in the rhizosphere and endosphere datasets.

Because total CH_4_ flux is a function of the activity of methanogenic vs. methanotrophic microbes, we next compared the relative abundance ratios of methanogenic archaea to methanotrophic bacteria in each plant species. The rhizosphere generally supported higher ratios of methanogens to methanotrophs compared to the endosphere (Fig. 3c). This is expected as roots contain the highest levels of oxygen in an otherwise flooded, anoxic environment and methanotrophs flourish under aerobic conditions (while the opposite is true for methanogens). We found that rice had a significantly higher ratio of methanogenic microbes than methanotrophic bacteria in both the rhizosphere and endosphere compared to native plants growing in the same environment. The native plants had mean ratios < 1 in both the rhizosphere and endosphere, while rice had mean ratios > 1 in both compartments. Without knowing the activity levels of methanogens and methanotrophs in our dataset, it is not possible to reach definitive conclusions regarding the efficiencies of rice and the native plants as methane producers or methane sinks. Nevertheless, these data are consistent with a primary role for the rice root microbiome in CH4 production from rice fields, as compared to those of the native plants.

### The rice core enriched microbiota show enrichment in domesticated soils

**Fig. 4:**
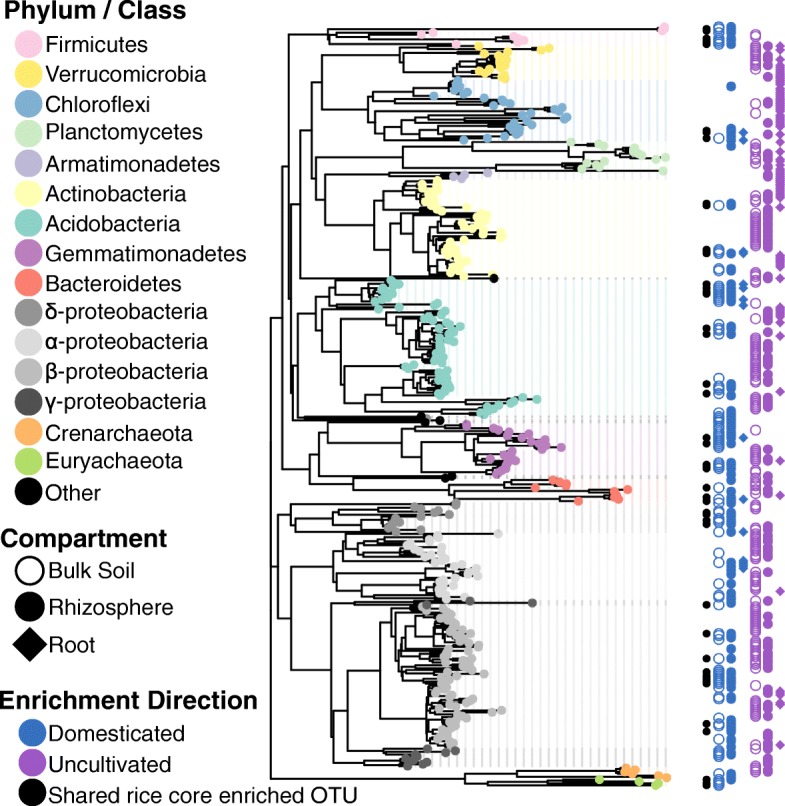
Soil domestication shifts the relative abundances of a taxonomically diverse set of OTUs. Phylogenetic tree displaying OTUs differentially abundant between uncultivated and domesticated soils. Colored points on the tips specify the phylum/class of a particular OTU. Colored points justified to the right of the tree indicate whether the OTU was significantly enriched in uncultivated (purple) or domesticated (blue) communities across compartments (indicated by the shape). Black points represent OTUs that overlap with the core set of rice enriched OTUs identified in Additional file 1: Figure S6

The above results suggest that rice plants acquire distinct root-associated microbiota compared to native plants growing in the same environment. Additionally, our results indicate that rice cultivation is associated with a considerable shift in soil microbiota from a wild status to a domesticated status. While flooding and nutrient addition likely play a role in domesticating rice field soils, we hypothesized that rice plants themselves are an important factor for domesticating soils via selective recruitment and exclusion of specific microbes. To support this hypothesis, we might expect there to be an overlap in domesticated soil enriched OTUs and rice core enriched OTUs. We compared the OTUs that were found to be significantly enriched in the microbiomes assembled from domesticated soils (Additional file 2: Table S6) to the rice core enriched microbes (Additional file 1: Figure S6, Additional file 2: Table S11). Of the 256 unique OTUs enriched in microbiomes originating from the domesticated soils, we found an overlap of 48 OTUs with the rice core enriched taxa (black data points, Fig. 4, Additional file 2: Table S12). This overlap was significantly greater than expected by chance given the contrasting microbiota between the two datasets (*P* = 1.88 × 10^−49^, hypergeometric test). Among the overlapping OTUs were two of the dominant methanogenic archaea taxa *Methanocella* and *Methanosarcina* as well as four OTUs within the genus *Anaerolinea*, which exhibits cooperative behavior when co-cultured with methanogens [27]. Conversely, only 8 rice enriched OTUs overlapped with the uncultivated soil enriched OTUs (*P* = 0.06, hypergeometric test). Of the native plant enriched OTUs, only one overlapped with the domesticated soil enriched OTUs and 12 overlapped with the uncultivated soil enriched OTUs (*P* = 1.41 × 10^−5^, hypergeometric test). The extent of the overlap between rice core enriched OTUs and domesticated soil OTUs is surprising given that the native plant experiment was conducted in Arkansas, USA, and the soils used for the soil domestication experiment were collected from locations in California. These results support the hypothesis that microbiota in domesticated field soils are shifted significantly by rice plants towards a composition that is characteristic for rice roots.

### Domesticated soils confer reduced rice seedling vigor compared to uncultivated soil

After establishing that soil cultivation history influences the composition of rice root-associated microbiota, we next investigated the impact of domesticated and uncultivated microbiota on seedling vigor traits in two independent experiments. A soil nutrient analysis showed differences in soil chemistry as a function of geography (Additional file 1: Figure S1B, C). Therefore, in order to avoid confounding edaphic abiotic and biotic factors (e.g., varying soil physical and chemical properties, potential allelopathic compounds, and other root metabolites), we grew rice plants in a common growth substrate inoculated with soil-derived microbiota suspensions. Furthermore, to confirm that the observed effects resulted from compositional differences rather than residual abiotic variation in our microbiota inocula, we grew plants in a substrate mock-inoculated with sterilized suspensions (see the “Methods” section).

**Fig. 5:**
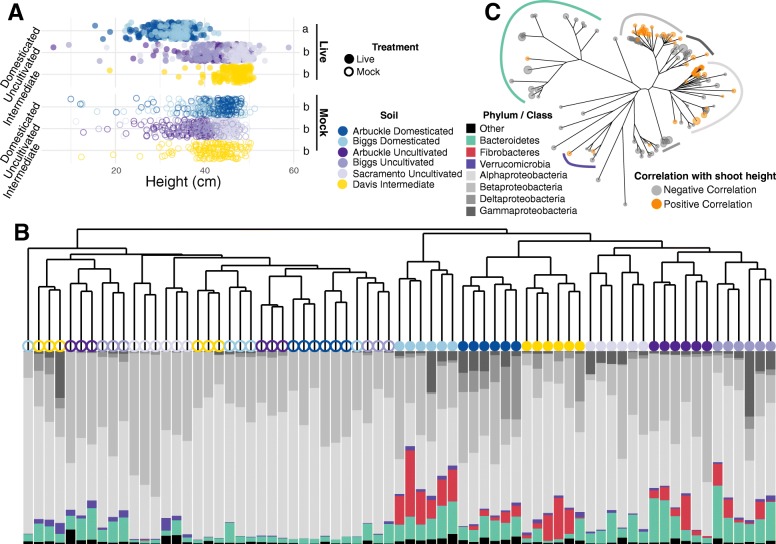
Compositional differences between domesticated and uncultivated soil communities correlate with differential plant growth in rice. **a** Heights of 21-day-old rice seedlings grown in calcined clay inoculated with live soil microbiota suspensions (solid circles) or mock suspensions (empty circles). Each color represents the history status and source of the soil used to generate the corresponding inoculum. Different letters specify significant differences between treatments (Tukey test, *P* < 0.05 after multiple-comparison adjustment). See also Additional file 1: Figure S9 for additional trait measurements. **b** Hierarchical clustering of endosphere communities based on Bray-Curtis dissimilarities between samples. The shape and color of each point represent inoculum type and soil source, respectively, and follow the same scheme as in panel **a**. The bar plot below each point displays the relative abundances of the most abundant phyla and Proteobacteria classes in each community. **c** Phylogenetic tree of endospheric OTUs significantly correlated with seedling height in plants grown in calcined clay inoculated with a live microbiota suspension (Wald test, *P* < 0.05 after multiple-comparison adjustment). The colored arcs indicate the phylum or Proteobacteria class of select branches and follow the same color scheme as in panel **b**

In the first experiment, inert calcined clay was inoculated with microbial communities derived from two domesticated soils and three uncultivated soils. Additionally, a sixth microbial inoculum was harvested from an experimental plot that cultivates rice during some summer seasons, while remaining fallow during others therefore representing an intermediate soil type. Rice seedlings growing with domesticated soil microbiota exhibited reduced shoot fresh weight and dry weight and height compared to plants associated with uncultivated and intermediate microbiota (Fig. 5a, Additional file 1: Figure S9A, Additional file 2: Table S13). Plants grown in mock-inoculated substrate displayed uniform shoot biomass and length, indicating that the differences exhibited between uncultivated and domesticated soil inocula are biological in nature (Fig. 5a, Additional file 1: Figure S9A, Additional file 2: Table S13).

In the second experiment, UC Mix III, a sandy plant growth matrix containing organic matter in the form of peat moss, was inoculated with two domesticated and two uncultivated soils. We again found that plants grown with live inocula differed significantly by soil cultivation history: plants which received inocula from domesticated soils had significantly reduced shoot fresh weight and shoot heights compared to plants which received uncultivated soil inocula (Additional file 1: Figure S9B, Additional file 2: Table S13). Plants which received mock inocula did not differ significantly by soil history status, again suggesting that the differences in seedling vigor traits that we witnessed between plants with domesticated and uncultivated soil microbiota were biological in nature (Additional file 1: Figure S9B, Additional file 2: Table S13).

We hypothesized that the divergence in the plant growth traits between soil types and inoculation types (live vs. mock) would correlate with microbiota structure; therefore, we next analyzed root-associated bacterial and archaeal microbiota for seedlings. Live and mock-inoculated seedlings hosted significantly divergent root microbiota in both experiments (Fig. 5b, Additional file 1: Figure S10, Additional file 2: Table S14): seedlings hosting the live inocula displayed significantly greater variation in microbiota structure compared to seedlings hosting mock inocula (Additional file 1: Figure S10), suggesting that filter sterilization/autoclaving was sufficient to disrupt microbiome structure. Seedlings inoculated with live soil communities also hosted microbiota which displayed increased separation between domesticated and uncultivated soils than plants hosting mock inocula (Additional file 1: Figure S10). Microbiota from seedlings inoculated with the intermediate soil type in experiment 1 clustered with the domesticated soil type microbiota (Fig. 5b) despite these plants displaying elevated seedling vigor characteristics (Fig. 5a). Together, these results indicated that differences between soil microbiota were reduced by filter sterilization/autoclaving and shows that divergences in seedling growth parameters correlate with microbiota structure.

We next sought to identify bacterial taxa whose relative abundance correlated with seedling vigor trait variation. We identified 151 OTUs which showed significant positive or negative correlations with seedling shoot height from experiment 1 plants inoculated with live soil microbiomes (Fig. 5c, Additional file 2: Table S15). Only 7 OTUs were identified showing significant positive or negative correlations with shoot height in seedlings hosting the mock communities, none of which overlapped with the live OTUs from live inoculations. Of the correlative OTUs of plants with live soil inoculations, 62 showed positive and 89 showed negative correlations, containing 4 and 9 phyla, respectively. OTUs with positive correlations to seedling height were largely composed of taxa belonging to Rhizobiaceae [22], Oxalobacteraceae [9], Comamonadaceae [6], and Methylophilaceae [4]. Negatively correlating OTUs were more taxonomically diverse, including 29 different bacterial families. Together, these results suggest that rice seedling vigor is negatively affected by microbes which accumulate over repeated seasons of cultivation.

## Discussion

Soils constitute a critical agricultural resource, and understanding how biotic components of the soils are impacted by crop cultivation and how, in turn, these changes affect crop performance will be important for sustained agricultural productivity. This study shows that the compositions of microbiota in soils from fields where rice has been cultivated for extended periods of time are considerably shifted from uncultivated, non-agricultural soils originating from geographically contiguous areas, which therefore potentially represent a pre-domesticated state. While cultivation practices, such as flooding and nutrient addition, are likely contributors to soil domestication, our findings suggest that rice plants themselves, through selective recruitment and diminishment of specific microorganisms, are also important drivers of the changes in microbiota during soil domestication (Fig. 4). Native plants growing in the same field environment do not appear to have a demonstrable role in shifting the soil microbiota towards a domesticated status, as these plants acquire microbiota distinct from the surrounding soil, from rice rhizosphere and roots, and from each other and are not prominent members of the rice field flora. Soil microbiota are influenced by plant cover [24, 25]; therefore, native plants may play a stronger role in rice field soil domestication as farmers use different weed control strategies.

We further characterized these changes in microbiota with respect to their impact on plant performance. Continuous rice cultivation under flooded conditions significantly shifts the soil microbiota in a rice field towards a more consistent microbial community structure (Fig. 1a, Additional file 1: Figure S2), which negatively impacts seedling vigor (Fig. 5a, Additional file 1: Figure S9) compared to uncultivated soil microbial inocula or sterilized inocula. This inhibitory effect is remarkably potent, as it can be observed with 200-fold dilutions of the soil microbiota inoculum into sterilized potting mix. Previous reports in rice have suggested that aerobically grown (i.e., not flooded) rice is susceptible to negative plant-soil feedback (also known as soil sickness), and have speculated that abiotic factors underlie the deleterious effect that continuous cultivation has on rice performance [19, 28]. Given the dilution factors of our inoculum, and the elimination of the effect after sterilization by filtration, we propose that biotic factors, specifically changes in the microbiota, are a major factor in the decline of plant vigor in domesticated soils. We consider unlikely the possibility that differences in seedling vigor could result from allelopathy, as rice plants display autotoxicity only when exposed to concentrations of root exudates greater than 100 mg/L [29], a concentration unachievable with our diluted inocula.

The mechanism of rice growth inhibition by microbiota in domesticated soil is presently unclear. A recent study in maize found growth inhibition by microbiota from agricultural soil growing wild-type corn plants at 10-fold dilutions, but not by microbiota from agricultural soil growing mutant corn deficient in production of DIMBOA, a metabolite important for herbivore defense [20]. Rice plants do not produce DIMBOA; therefore, DIMBOA exudation cannot explain the observed inhibition of growth by rice field microbiota, which we find to be effective even at much higher dilutions. These observations imply that plant-soil feedback is a general outcome of crop cultivation, in which multiple mechanisms are likely to be involved. From our study, it is not possible to determine the number of growing seasons necessary to domesticate soils such that they have negative impacts on seedling vigor. However, we do show that the rhizosphere microbiota of plants grown in uncultivated soil show greater similarity to rhizosphere microbiota of rice grown in domesticated soils, than to the microbiota of unplanted domesticated soils and uncultivated soils (Fig. 1b). These data suggest that soil domestication has already initiated at 6 weeks after germination and is presumably reinforced by multiple seasons of cultivation. We further found that seedlings with soil inocula from a rice field left fallow for over a growing season hosted microbiota more similar to domesticated soils (Fig. 5b, Additional file 1: Figure S10). Unexpectedly, these seedlings displayed vigor traits equal to or greater than uncultivated soils (Fig. 5a, Additional file 1: Figure S9A). These results suggest that the negative effects of continuous rice cultivation imparted by microbiota may be reversible if rice cultivation is halted even temporarily.

The growth inhibition observed in our study does not appear to arise from specific prokaryotic taxa. Negative correlation with seedling height was widely distributed across bacterial phyla and classes. However, positive correlation with growth was more restricted in distribution and included several taxa within the order Rhizobiales. Specifically, we identified 13 *Rhizobium* OTUs, 4 Agrobacterium OTUs, and 2 *Devosia* OTUs that correlated positively with seedling height. Rhizobiales are widely distributed in natural soils, a pattern also observed in a recent study of native prairie soils relative to cultivated maize plots, although possible correlations with plant vigor and negative plant-soil feedback were not examined [21]. Interestingly, a recent study found that Rhizobiales bacterial isolates generally induced growth promotion in *Arabidopsis thaliana* and that some *Rhizobium* strains interfered with the MAMP-triggered immunity response, perhaps allowing for root colonization without causing a negative effect on plant growth by induction of an immune response [30]. These results suggest that rice soil domestication selects for an enrichment of microbes deleterious for plant growth at the expense of growth-promoting bacteria (Additional file 1: Figure S11). We did not examine the impact that soil domestication may have on the fungal communities, and therefore, we cannot exclude that the feedback effect on rice growth arose from specific fungal taxa. However, a study found that peanut plants grown in field soil subjected to monoculture show upregulated expression of genes involved in defense against bacteria but not fungi, suggesting that at least in that system, bacterial communities are responsible for the deleterious effects on plant growth [16]. An implication of this inference is that partial remediation of such negative effects might be feasible through growth-promoting microbes supplied to plants grown in domesticated soil. It is interesting to note that major shifts in human and captive nonhuman primate gut prokaryotic microbiota have been shown to be correlated with diets typical of industrialized societies [31–34]. Despite likely differences in the specific mechanisms, they illustrate a similar underlying concept in which industrialization and development can lead to unintended consequences through modulation of microbiomes.

The results from this study also have implications for agriculture-related production of greenhouse gases. Paddy fields account for 15–30% of anthropogenic methane emissions [35, 36]. Since methane has a greenhouse warming potential that is 25-fold greater than carbon dioxide [37], anticipated increases in rice cultivation to meet future demand make it important to understand the potential impacts on methanogens. Flooded soils, including marshlands, maintain anaerobic conditions that are known to favor methanogenic archaea [26, 38]. However, in addition to the anoxic environment imposed by flooding, it is not clear whether methanogen residence in rhizosphere and root tissues exhibit plant host-specific enrichment. Here we have shown that specific methanogenic archaea are uniquely enriched in the rhizosphere and roots of rice plants compared to native plants growing in the same flooded environment. Furthermore, methanogenic archaea are also enriched in microbiota of rice plants grown in rice domesticated soils compared to wild soils (Fig. 4). These data suggest the preponderance of some dominant methanogenic archaea in rice fields might be facilitated through a two-step process. Flooded conditions provide favorable anaerobic conditions for methanogen establishment, thus setting the stage for colonization of the rhizosphere and root tissue of the rice plants. Rice plants then enhance colonization of specific methanogens, as compared with other aquatic native plants that appear to not support methanogen entry to the rhizosphere and endosphere (Additional file 1: Figure S8). Previous studies have indicated that the archaea *Methanocella* is a predominant utilizer of rice plant-derived carbon [39, 40], and its genome encodes pathways for carbon assimilation as well as aerotolerance [41]. In our study, *Methanocella* and *Methanosarcina*, another dominant methanogen in rice soils, were found to be enriched in domesticated soils compared to uncultivated soils, and both were also present in the set of rice core enriched microbes (Fig. 4). Thus, an important byproduct of soil domestication by rice cultivation is the buildup of methanogenic archaea that could have longer term climatic consequences. If rice is a strong driver of highly active methanogens, then this accumulation might be ameliorated by an imposed discontinuity of rice cultivation within a field, or by selecting rice cultivars that are lower in methane emissions [42] that might be potentially less active in methanogen recruitment and growth.

## Conclusion

This study characterizes the consequences of domestication on soil microbiomes and on plant-soil feedback arising from continuous monoculture of rice, globally the most important food crop. The findings indicate that compositional shifts in the soil microbiota appear to be partly driven by the rice plants and are not solely a consequence of cultivation practices. These microbiota changes can inhibit plant growth and potentially impact agricultural yields, as well as contribute to global methane emissions. Questions that should be addressed in future research will include the extent of persistence of the altered microbiota with crop rotation, or if cultivation is paused or terminated, the rate of decay of the domesticated state in the absence of a feedback loop, and the degree of reversion to the microbiota compositions of the geographically related uncultivated soils.

## Methods

### Soil collection and characterization

Soils used in the soil domestication and seedling vigor studies were collected from multiple sites across the California Central Valley (Additional file 1: Figure S1A). Domesticated soils were harvested from rice fields in Arbuckle (39° 00′ 42.2″ N, 121° 55′ 19.6″ W) and Biggs (39° 27′ 50.8″ N, 121° 44′ 14.4″ W); uncultivated soils were harvested from non-agricultural sites in Arbuckle (39° 00′ 44.8″ N 121° 53′ 09.4″ W), Biggs (39° 27′ 53.0″ N 121° 43′ 49.9″ W), and Sacramento (38° 34′ 29.6″ N 121° 38′ 43.8″ W); and one intermediate soil was harvested from an experimental plot in Davis (38° 32′ 37.9 N, 121° 48′ 44.0″ W). Soil chemistry profiling was performed by the UC Davis Analytical Laboratory.

### Soil domestication study

**Fig. 6:**
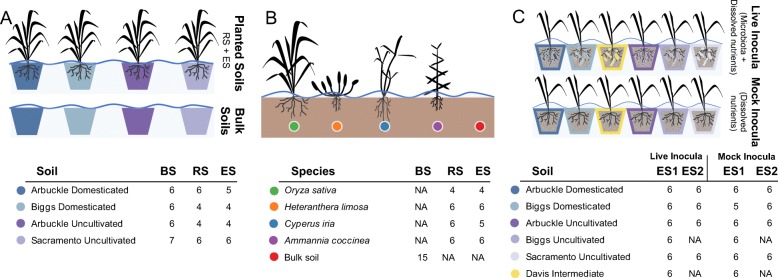
Experimental design. **a** Soil domestication study: rhizospheres and endospheres of rice plants grown in two domesticated and two uncultivated soils were 16S rRNA gene profiled to understand how soil cultivation history affects root microbiome assembly. Additionally, unplanted bulk soils were sampled to characterize the inherent compositional differences between soil types. Both planted and unplanted potted soils were kept under submergence in a controlled greenhouse setting. **b** Native plant study: rhizospheres and endospheres of rice (*Oryza sativa*), mudplantain (*Heteranthera limosa*), sedge (*Cypeus iria*), and redstem (*Ammania coccinea*) were 16S rRNA gene profiled to explore differences between a monoculture crop and native plant species grown in the same flooded rice paddy. Additionally, bulk soil samples were collected to understand the compositional relationship of their associated communities to those acquired by the conspecific plant (rice) and the other hosts. **c** Seedling vigor study: rice plants were grown in a common substrate treated with microbial inocula derived from domesticated, uncultivated, or intermediate soils in order to analyze the effect of soil microbiomes with distinct cultivation histories on plant growth. As a control, plants were grown in substrate treated with corresponding sterilized inocula in order to account for any residual abiotic variation. Additionally, the endospheres of a subset of plants were 16S rRNA gene profiled to assess community structure. In all panels, tables under each graphic represent the number of replicates sequenced for each treatment/sample type combination. BS, RS, and ES stand for bulk soil, rhizosphere, and endosphere communities, respectively; ES1 and ES2 represent endosphere communities collected from the first or second seedling vigor experiment, respectively. In all cases, NA stands for not applicable

This study was conducted in two batches using four different soils (Additional file 1: Figure S1A). The first batch included an uncultivated soil from Sacramento and a domesticated soil from Arbuckle (both collected on April 10, 2015), while the second batch included an uncultivated soil from Arbuckle and a domesticated soil from Biggs (both collected on June 3, 2016). Soils were homogenized, placed into pots, and kept under submerged conditions with deioinized water in controlled greenhouse conditions (Fig. 6a). Half the pots were reserved for unplanted soil controls, and the other half were used to transplant 7-day-old axenic rice seedlings (cultivar M206) germinated in 0.5× MS agar plates from surface-sterilized dehulled seeds (70% bleach for 5 min, followed by three washes in autoclaved deionized water). The plants and soils were irrigated under flooded conditions for the duration of the experiments. Plants and soils were supplemented with nutrient solution every 14 days. Six weeks after transplantation, samples were harvested and bulk soil, rhizosphere, and endosphere communities were immediately processed following the steps described below.

### Seedling vigor study

This study encompassed two independent experiments, each one with specific growth substrates, inoculation treatments, and harvesting times (Fig. 6b).

#### Experiment 1

This study included six different soils: two domesticated soils from Arbuckle and Biggs; three uncultivated soils from Arbuckle, Biggs, and Sacramento; and the intermediate Davis soil (all collected on April 5, 2018). Each soil was homogenized, scooped into a pot, and kept under submerged conditions for 10 days. Soil suspensions were then generated by stirring 100 g of submerged soil into 1 L of sterile 0.5× strength MS media. For the live microbiota treatments, 500 mL of each soil suspension was added to 10 L of twice-autoclaved calcined clay. The inoculated substrate was thoroughly mixed and scooped into two 72-cell propagation trays that were immediately bottom-saturated with deionized water to achieve a submerged condition. For the mock inoculation treatments, the same procedure was followed except soil suspensions were allowed to settle for 30 min before collecting and filter-sterilizing (0.22-μm filter membrane, Millipore Sigma, SCGPU10RE) the supernatant. Surface-sterilized hulled rice seeds (10% bleach for 1 h, followed by three washes in autoclaved deionized water) were then sewn into the inoculated calcined clay. For each of the 12 treatment/soil combinations, 144 seeds were planted. Plants were kept under controlled greenhouse conditions and bottom-irrigated to maintain submerged conditions. Upon harvesting, the shoot height and fresh weight of 21-day-old rice seedlings were immediately registered. The collected tissue was then allowed to dry for 1 week before measuring the dry weight. Additionally, whole root systems (three per tray, six per treatment/soil combination) were collected in sterile PBS for endosphere microbiome characterization following the steps described below.

#### Experiment 2

This study included four different soils: two domesticated soils from Arbuckle and Biggs and two uncultivated soils from Arbuckle and Sacramento (all collected during November 2016). Each soil was homogenized, scooped into a pot, and kept under submerged conditions for 14 days. For the live microbiota treatments, 18 g of submerged soil stirred into 1 L of sterile 0.5× strength MS media was added to 1.8 kg of twice-autoclaved UC Mix III. UC Mix III is a potting soil mix utilized by University of California campuses that is primarily composed of sand and peat moss (https://agops.ucr.edu/soil/). For each soil, the inoculated substrate was thoroughly homogenized and scooped into 16 8-cell polypropylene boxes previously perforated to allow water flow. The boxes were then evenly distributed between two plastic trays and bottom-saturated with deioinized water. For the mock inoculation treatments, the same procedure was followed except soil suspensions were autoclaved before inoculating the UC Mix III substrate. Surface-sterilized hulled rice seeds (1% bleach for 2 h, followed by three washes in autoclaved deionized water) were then sewn into the inoculated UC mix III. For each of the eight treatment/soil combinations, a total of 256 seeds were planted (2 seeds per well within each cell of the polypropylene box), and later thinned to 128 seedlings per treatment. Plants were kept under controlled greenhouse conditions and bottom-irrigated to maintain submerged conditions. Upon harvesting, the shoot height and fresh weight of 14-day-old rice seedlings were immediately registered. Additionally, whole root systems (three per tray, six per treatment/soil combination) were collected in sterile PBS for endosphere microbiome characterization following the steps described below.

### Native plant study

Rice (*Oryza sativa*, cultivar Sabine), valley redstem (*Ammania coccinea*), blue mudplantain (*Heteranthera limosa*), and flatsedge (*Cypeus iria*) plants (*n* = 4–6 per host) were harvested in a flooded paddy near Jonesboro, Arkansas, on August 22, 2015 (Fig. 6c). Roots were collected from plants in the reproductive stage as plant phenology affects the root microbiota composition [4, 23, 43]. Roots were vigorously shaken to remove loose soil and collected into 50-mL Falcon tubes with 15 mL of sterile PBS solution. Additionally, unplanted bulk soils (*n* = 15) were directly collected into 50-mL Falcon tubes. All samples were immediately stored on ice and shipped overnight to the University of California, Davis. Upon receiving them, bulk soil, rhizosphere, and endosphere compartments were processed for DNA extraction as described below [44]. Briefly, harvested rice roots were vigorously shaken to remove loosely bound soil and collected into 50-mL Falcon tubes with 15 mL of sterile PBS solution. Rhizosphere fractions were then harvested by vortexing the roots and collecting 500 μL of the resulting soil slurries into PowerBead tubes for DNA extraction. Roots were then vortexed in consecutive washes of fresh PBS solution until all soil was depleted and sonicated three times at 50 Hz for 30 s in fresh PBS to remove all rhizoplane microorganisms. The remaining roots were then placed into PowerBead tubes for endosphere DNA extraction. For bulk soil samples, ~ 250 mg of soil was directly placed into PowerBead tubes for DNA extraction. All DNA extractions were performed using the MoBio Powersoil DNA isolation kit.

### 16S rRNA gene amplification and sequencing

All 16S rRNA gene amplification was performed as noted in [44]. Briefly, the V4 region of the 16S rRNA gene was amplified using PCR with a dual indexing strategy. For each PCR reaction, a corresponding negative control was also performed. All reactions were checked for amplification by running PCR products out on a 1% agarose gel. If a reaction’s negative control succeeded in amplification, then we discarded the particular reaction and reperformed the PCR. The PCR reactions were purified using AMPure beads and measured for concentration using a Qubit. The PCR products were pooled in equimolar concentrations, concentrated using AMPure beads, and then gel extracted from a 2% agarose gel. Sequence libraries were sent to the University of California DNA Technologies Core Laboratory for 250 × 250 bp sequencing on the Illumina Miseq platform.

### Sequence processing

The resulting paired end sequences were demultiplexed using custom Python scripts [44] and aligned into contiguous reads using PANDAseq [45]. The contiguous reads were discarded if containing any ambiguous bases or if the length exceeded 275 bases. All reads were then clustered into OTUs based upon 97% sequence identity using NINJA-OPS [46]. OTUs with plastid and mitochondrial taxonomies were removed from all resulting OTU tables.

### Statistical analyses

All statistical analyses were conducted using R version 3.1 [47]. Unless otherwise noted, we determined statistical significance at *ɑ* = 0.05 and, where appropriate, corrected for multiple hypothesis testing using the Benjamini and Hochberg method [48]. For beta-diversity analyses, OTU counts were normalized using the variance-stabilizing transformation implemented in DESeq2 [49, 50]. Shannon diversity was calculated using the diversity() function, PCoA and CAP analyses were conducted using the capscale() function, perMANOVA was conducted using the adonis() function, distances to within-group centroids were calculated (i.e., Additional file 1: Figure S3) using the betadisper() function, and Bray-Curtis dissimilarities were calculated using the vegdist() function all from the Vegan package [51]. Differential abundance analyses were performed with the DESeq2 package [49, 50]. Linear mixed effects models were fit with the lmerTest package [52]. Beta regression was run using the betareg() function from the betareg R package [53], and ANOVA was run using the aov() function the Stats package [47]. Hypergeometric tests were run using the phyper() function. Phylogenetic trees were displayed using the plot_tree() command from the PhyloSeq package [54]. All other graphs and plots were generated using the ggplot2 package [55].

## Supplementary information

**Additional file 1: Figure S1.** Collection sites of domesticated and uncultivated soils. **Figure S2.** Weighted and Unweighted Unifrac dissimilarity metrics reveal differing microbiota acquired by rice plants grown in flooded domesticated and uncultivated soils. **Figure S3.** Uncultivated communities are more variable than domesticated communities across compartments. **Figure S4.** Taxonomic classification of OTUs differentially abundant between soil cultivation histories. **Figure S5.** Weighted and unweighted Unifrac dissimilarity metrics reveal that root compartment and domestication history affect community composition. **Figure S6.** Compositional differences between rice and native plants stem from the simultaneous enrichment and depletion of several OTUs. **Figure S7.** Taxonomic spread of OTUs consistently enriched or depleted the rhizosphere and endosphere communities of rice. **Figure S8.** Methanogen relative abundance differs between bulk soil samples and rhizospheres of rice and native plants - but in opposite directions. **Figure S9.** Domesticated soil microbiota reduces plant growth in rice. **Figure S10.** Endosphere communities are structured by soil source in rice plants inoculated with a live microbial suspension. **Figure S11.** Simplified model describing soil domestication by rice cultivation and its impact on rice root-associated microbiota as well as seedling vigor.

**Additional file 2 : Table S1.** Soil chemistry profiles from domesticated and uncultivated soils used in the domestication experiment. **Table S2**. Permanova statistics on Bray-Curtis dissimilarities of the soil domestication study. **Table S3.** Permanova statistics on weighted and unweighted unifrac dissimilarity metrics for soil domestication study. **Table S4.** Betadispersion estimates for the soil domestication study. **Table S5.** Results of testing for differential abundance in each compartment between domesticated and uncultivated soils. **Table S6.** Differentially abundant OTUs between Domesticated and Uncultivated soils. **Table S7.** Permanova statistics on Bray-Curtis dissimilarities for the native plants study. **Table S8.** Permanova on weighted and unweighted Unifrac dissimilarity metrics for the native plants study. **Table S9.** Tukey HSD results for testing dissimilarity metrics for the rhizosphere microbiome of each plant species to bulk soil. Rice show significantly less distance to bulk soil than other plant species. **Table S10.** Tests for differential abundance to rice microbiota for each compartment and each plant host species. **Table S11.** Taxa that belong to the rice core enriched and rice core depleted microbes. **Table S12.** Overlapping OTUs enriched in domesticated soil microbiota and rice core enriched microbiota. **Table S13.** Post hoc tests comparing seedling vigor traits between soil inoculum types. **Table S14.** Permanova statistics on Bray-Curtis dissimilarities from the seedling vigor experiments. **Table S15.** OTUs showing significant correlations with seedling height in Seedling Vigor Experiment 1.

**Additional file 3.** Review history.

## Data Availability

Raw sequences can be accessed in the Short Read Archive of NCBI under project no. PRJNA548898 [56]. All processed datasets have been deposited in a Zenodo repository 10.5281/zenodo.3372822 [57]. R notebooks for the full analyses are freely available under the GNU General Public License v3.0 in the GitHub repository https://github.com/bulksoil/SoilDomestication [58].
