# Supplementary material for: Soil domestication by rice cultivation results in plant-soil feedback through shifts in soil microbiota
Source: Genome Biol. 2019 Oct 24;20:221. doi: 10.1186/s13059-019-1825-x (PMC6814045; doi:10.1186/s13059-019-1825-x)
Supplement: Supplementary file 1 — Additional file 1: Figure S1. Collection sites of domesticated and uncultivated soils. Figure S2. Weighted and Unweighted Unifrac dissimilarity metrics reveal differing microbiota acquired by rice plants grown in flooded domesticated and uncultivated soils. Figure S3. Uncultivated communities are more variable than domesticated communities across compartments. Figure S4. Taxonomic classification of OTUs differentially abundant between soil cultivation histories. Figure S5. Weighted and unweighted Unifrac dissimilarity metrics reveal that root compartment and domestication history affect community composition. Figure S6. Compositional differences between rice and native plants stem from the simultaneous enrichment and depletion of several OTUs. Figure S7. Taxonomic spread of OTUs consistently enriched or depleted the rhizosphere and endosphere communities of rice. Figure S8. Methanogen relative abundance differs between bulk soil samples and rhizospheres of rice and native plants - but in opposite directions. Figure S9. Domesticated soil microbiota reduces plant growth in rice. Figure S10. Endosphere communities are structured by soil source in rice plants inoculated with a live microbial suspension. Figure S11. Simplified model describing soil domestication by rice cultivation and its impact on rice root-associated microbiota as well as seedling vigor. [file 13059_2019_1825_MOESM1_ESM.pdf]

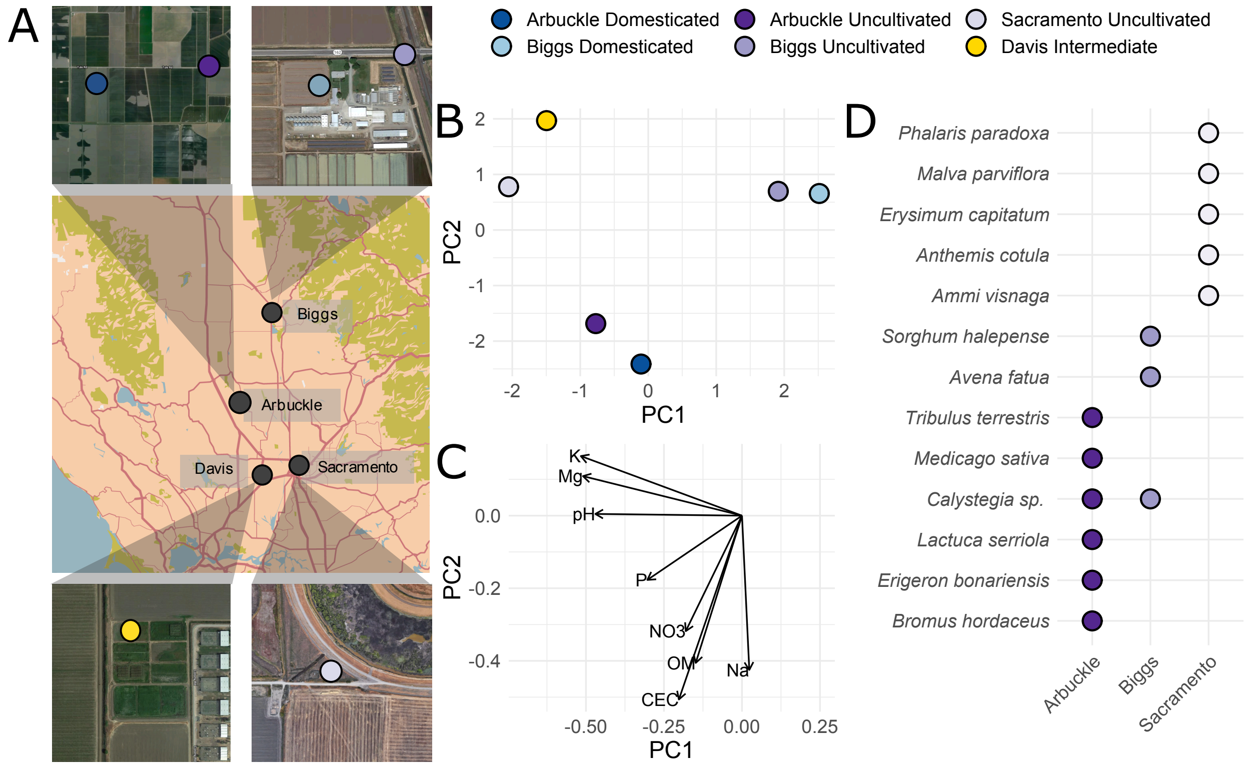

Figure S1. Collection sites of domesticated and uncultivated soils. (A) Map of the California Central Valley displaying the collection sites for all of the soils used in the domestication and seedling vigor studies. (B-C) Scores (B) and loadings (C) of a principal component analysis performed on soil nutrient profiles. (D) List of representative plant species found in each of the sites where uncultivated soils were collected.

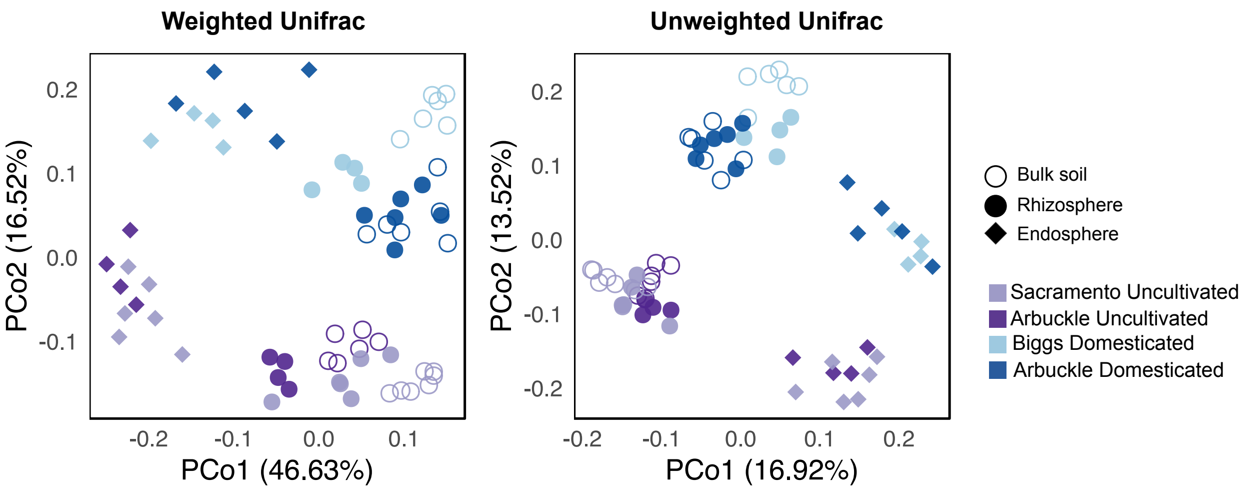

Figure S2. Weighted and Unweighted Unifrac dissimilarity metrics reveal differing microbiota acquired by rice plants grown in flooded domesticated and uncultivated soils.

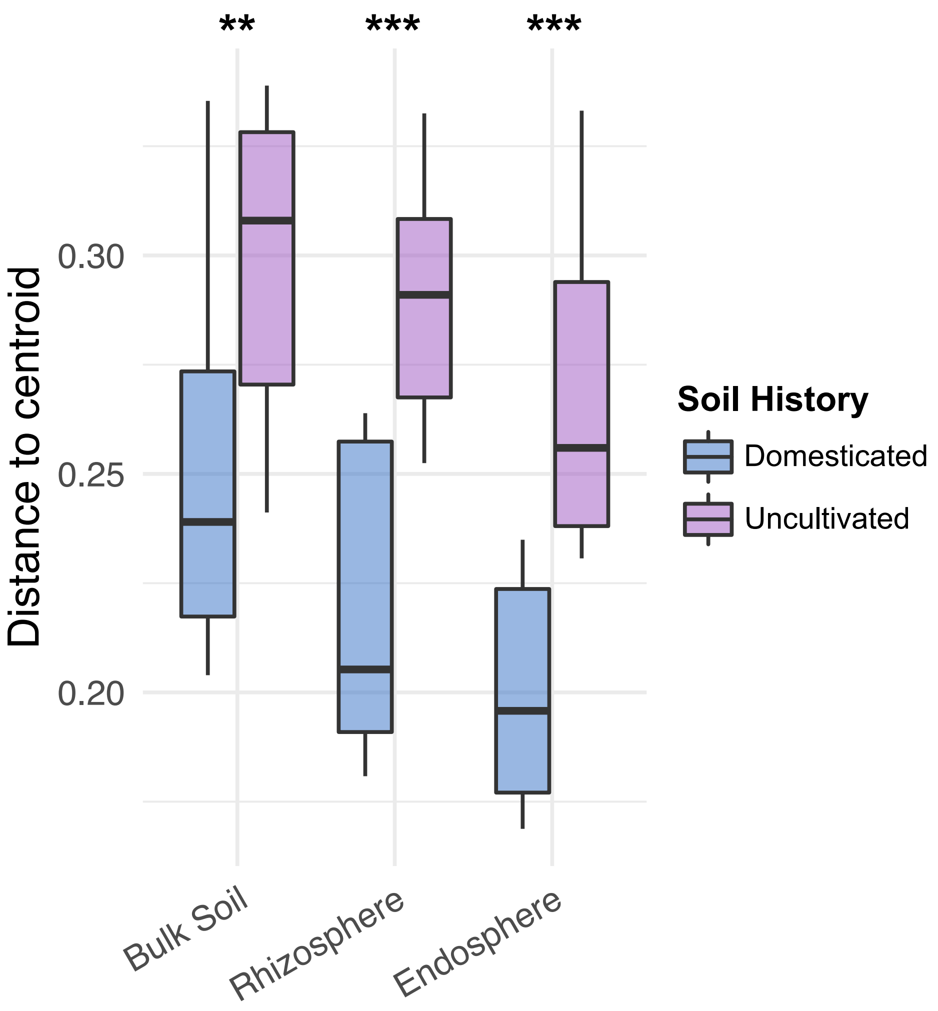

Figure S3. Uncultivated communities are more variable than domesticated communities across compartments. Distances of bulk soil, rhizosphere, and endosphere samples to their respective PCoA centroids within each soil history status. Principal coordinate analysis was performed using Bray-Curtis dissimilarities. Asterisks indicate significant differences (Tukey's Honest Significant Differences test, ** $P < 0.01$, *** $P < 0.001$).

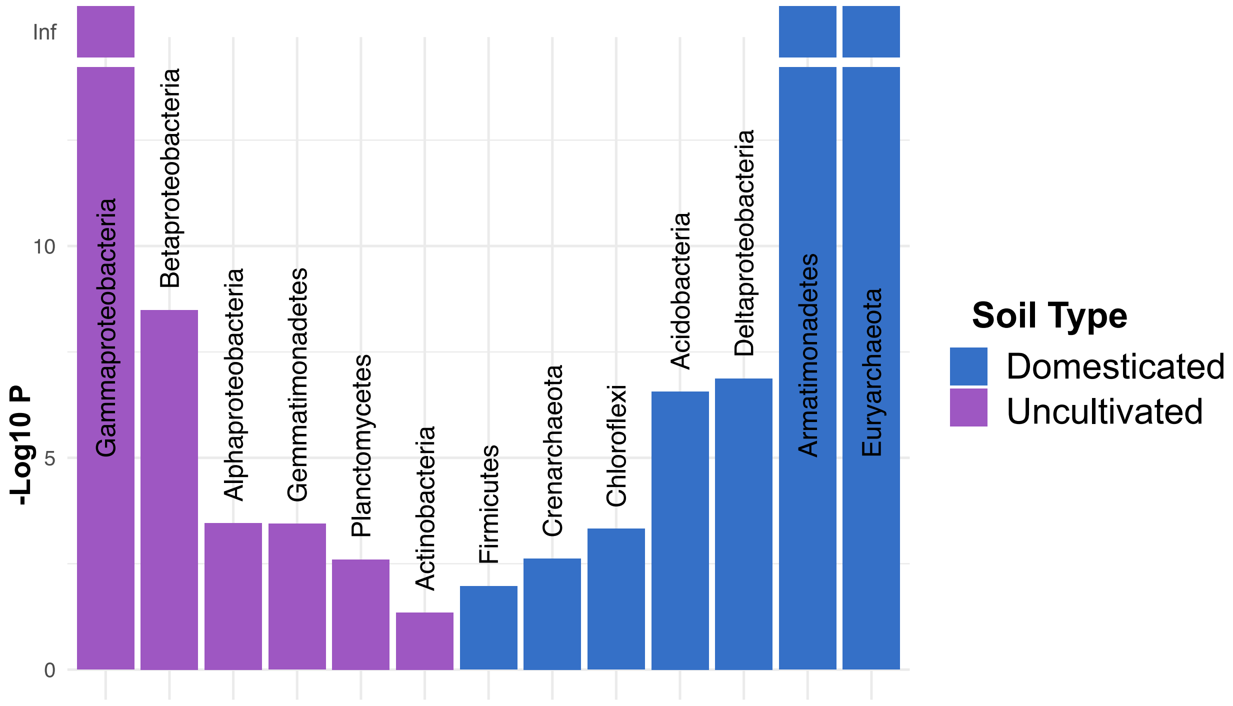

Figure S4. Taxonomic classification of OTUs differentially abundant between soil cultivation histories. Enrichment factors for specific phylum level classification of OTUs found to be significantly enriched or depleted in domesticated soils relative to uncultivated soils. Enrichment factors were calculated as -log10(P), where P is the probability that this phylum is represented more in the specific class more than expected by random chance (hypergeometric test).

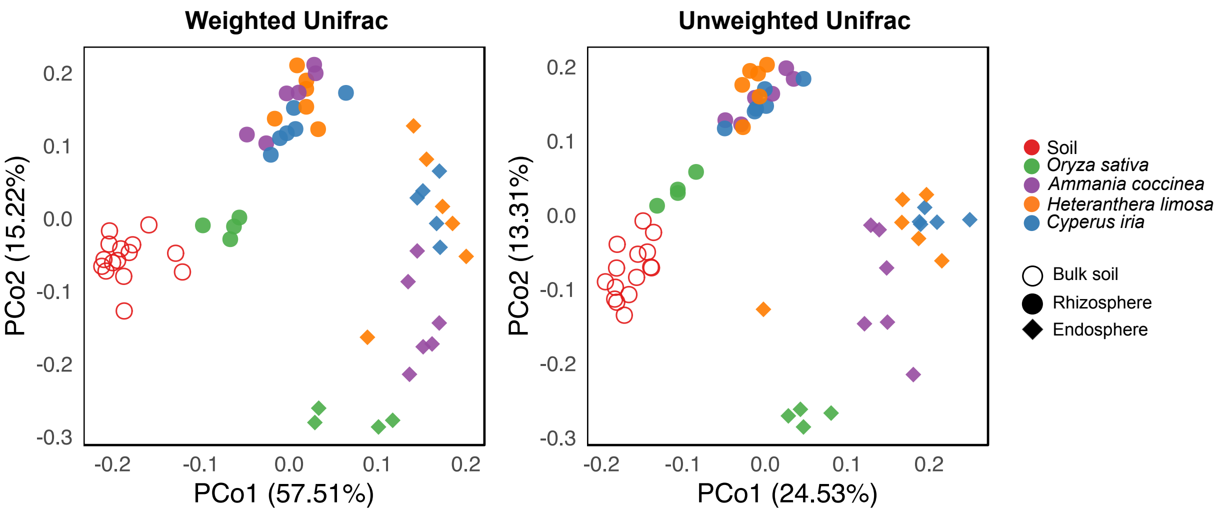

Figure S5. Weighted and unweighted Unifrac dissimilarity metrics reveal that root compartment and domestication history affect community composition.

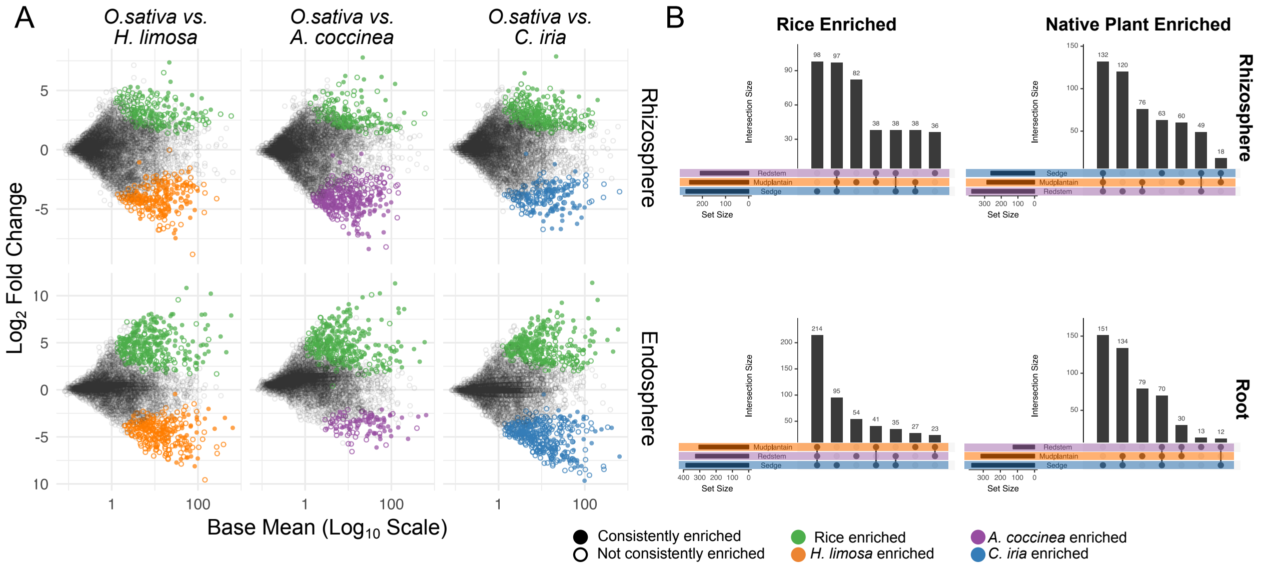

Figure S6. Compositional differences between rice and native plants stem from the simultaneous enrichment and depletion of several OTUs. (A) Patterns of differential OTU abundance between rice and native plants grown in flooded paddy fields. The x-axis represents the mean relative abundance of each OTU and the y-axis indicates its log2 fold change relative to a specific native plant: a positive value indicates enrichment in rice plants and a negative value indicates enrichment in the native plant species. Colored points correspond to OTUs significantly affected by plant host (Wald test, $P < 0.05$ after multiple comparison adjustment) and solid points highlight OTUs that are consistently affected across all pairwise comparisons. (B) Intersection of the sets of differentially abundant OTUs detected in the pairwise comparisons between rice and the three native plant species.

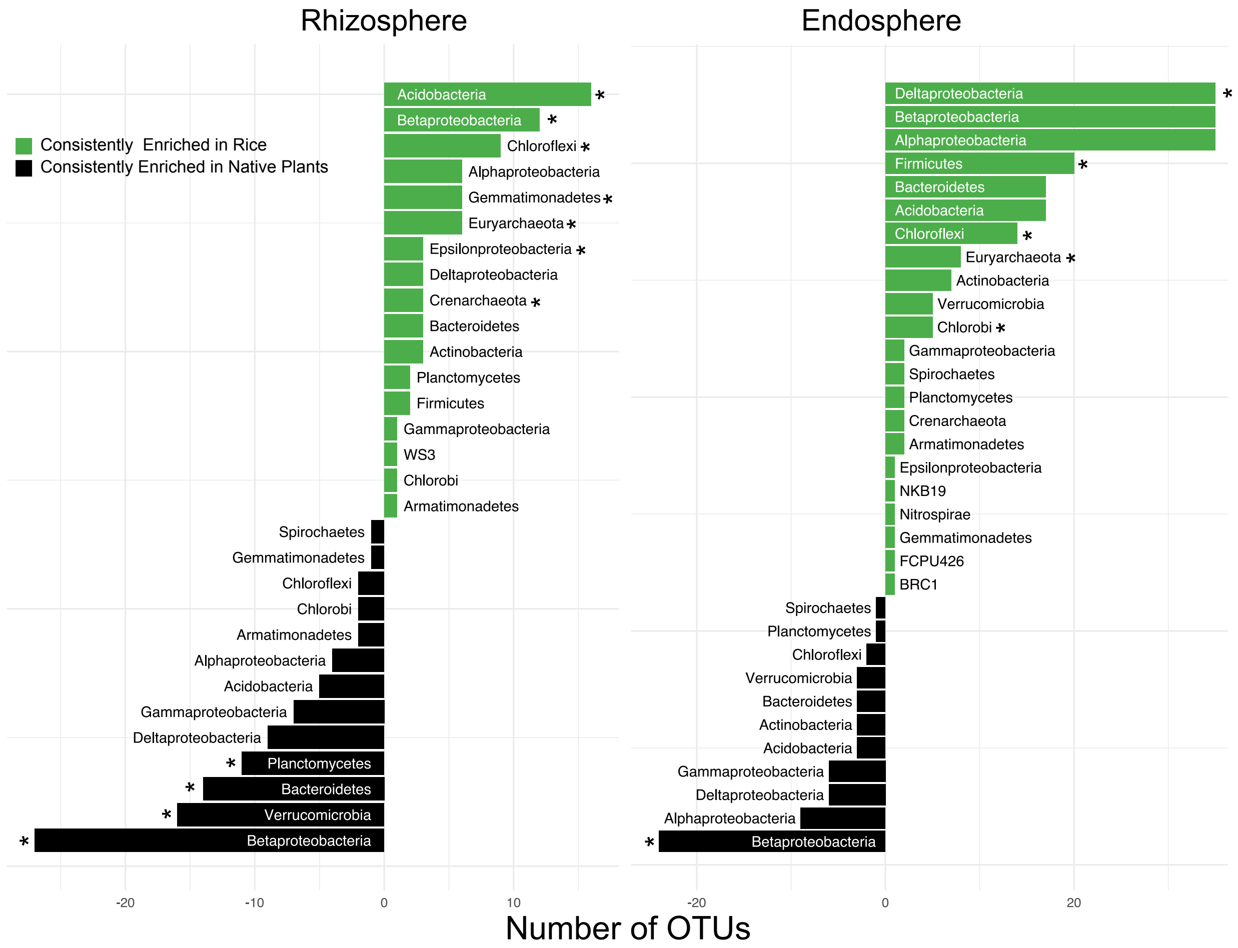

Figure S7. Taxonomic spread of OTUs consistently enriched or depleted the rhizosphere and endosphere communities of rice. Number of OTUs that belong to a particular phylum / Proteobacteria class in the core sets of OTUs consistently enriched (green) or depleted (black) in rice root communities relative to native plant communities. Asterisks indicate taxa with a significant overrepresentation of differentially abundant OTUs (hypergeometric test, $P < 0.05$).

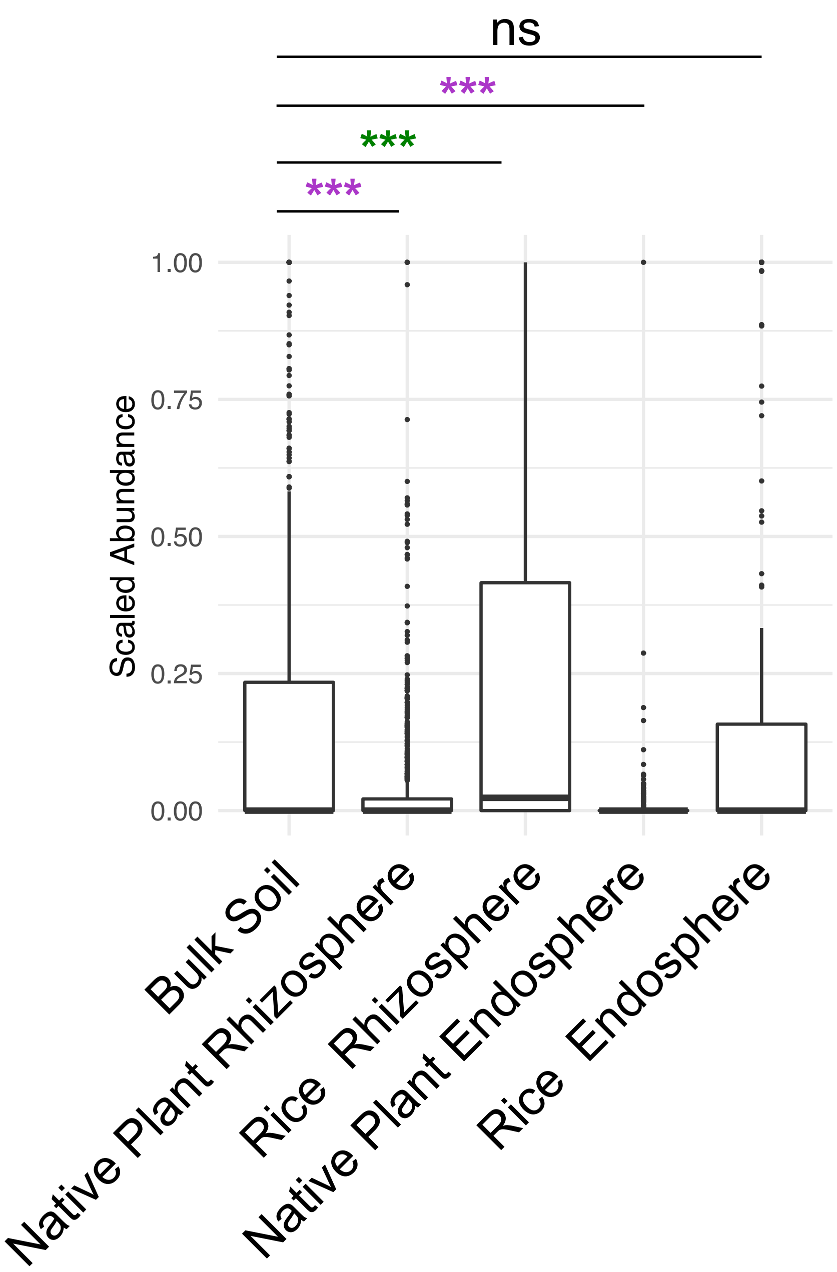

Figure S8. Methanogen relative abundance differs between bulk soil samples and rhizospheres of rice and native plants - but in opposite directions. Scaled relative abundance values for each methanogen in each sample are plotted as a distribution. Purple asterisk indicate that the relative abundance of methanogens is significantly greater in bulk soil samples. Green asterisks indicate that relative abundance of methanogens is greater in rice samples. Statistical significance was calculated using Tukey's Honest Significant Differences test on an ANOVA model. *** indicates adjusted P values were less than 0.005.

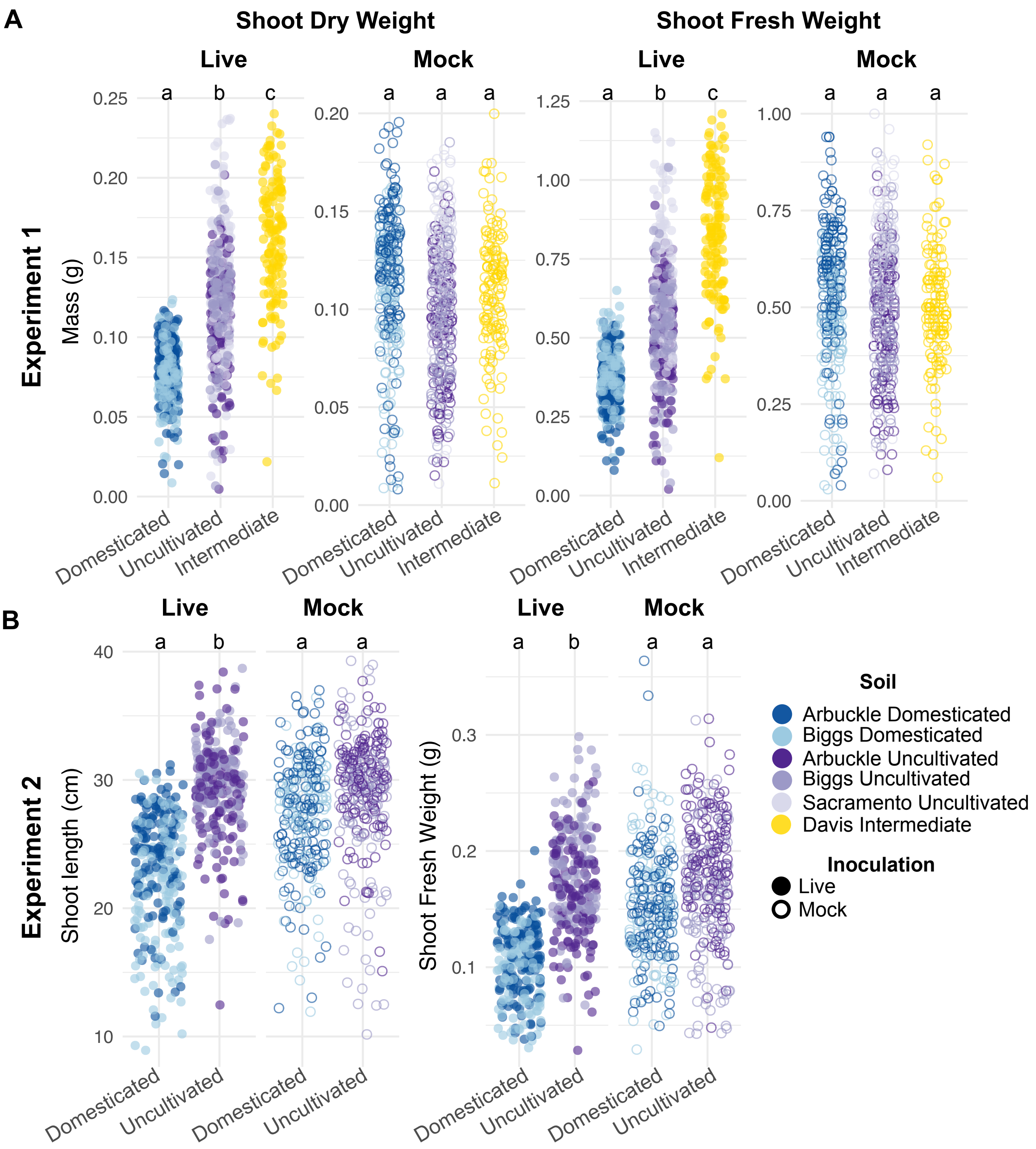

Figure S9. Domesticated soil microbiota reduces plant growth in rice. (A) Dry and fresh shoot weights of 21-day old rice seedlings grown in calcined clay inoculated with live soil- microbiota suspensions (solid circles) or mock suspensions (empty circles). (B) Height and fresh shoot weight of 14-day old rice seedlings grown in UC mix inoculated with live soil-microbiota suspensions (solid circles) or mock suspensions (empty circles). In both A and B, each color represents the history status and source of the soil used to generate the corresponding inoculum. Different letters specify significant differences between treatments (Tukey test, P < 0.05 after multiple-comparison adjustment).

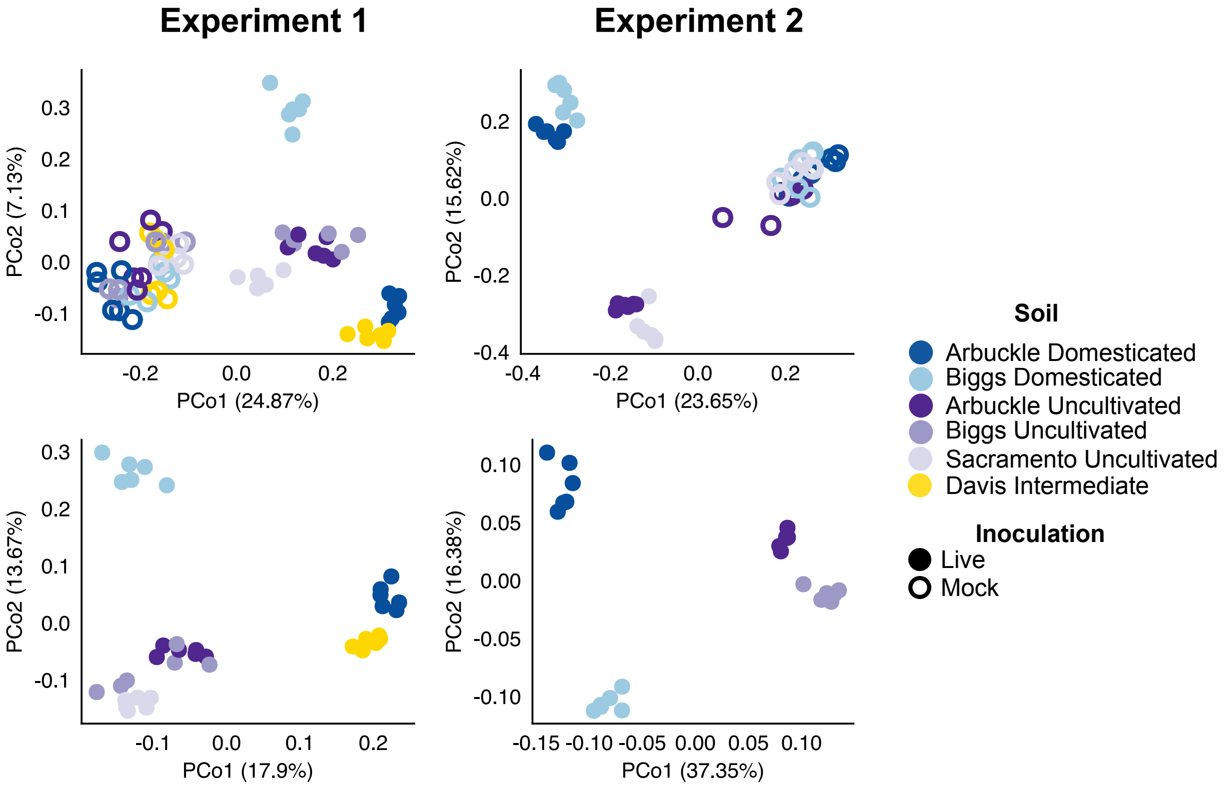

Figure S10. Endosphere communities are structured by soil source in rice plants inoculated with a live microbial suspension. Principal coordinate analysis of endosphere communities of rice plants grown in calcined clay (Experiment 1) or UC Mix (Experiment 2) inoculated with live soil-microbiota suspensions (solid circles) or mock suspensions (empty circles). In the bottom panels mock-inoculated samples were excluded to better display the differences between soil types in the live-inoculated communities. In all panels, each color represents the history status and source of the soil used to generate the corresponding inoculum.

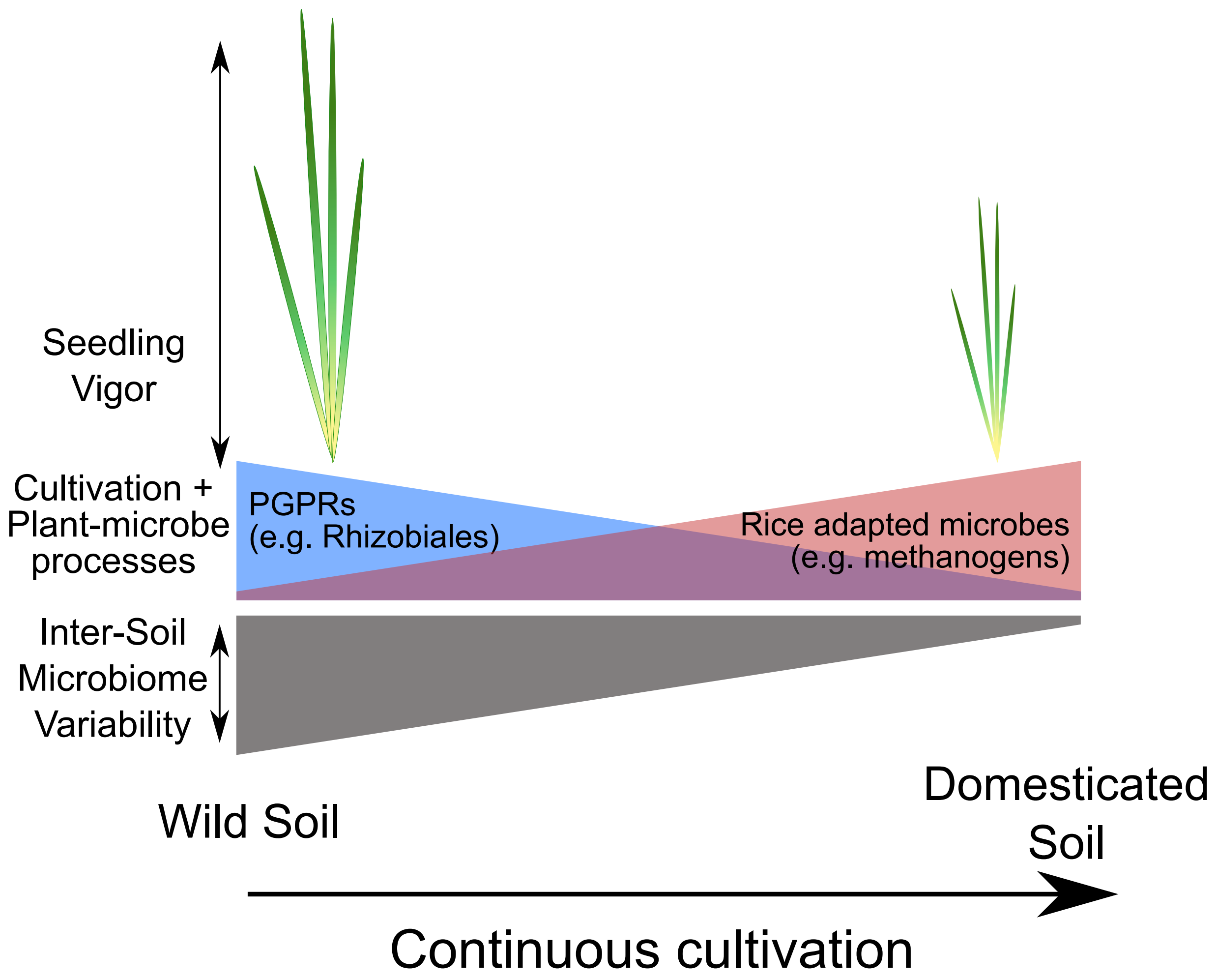

Figure S11. Simplified model describing soil domestication by rice cultivation and its impact on rice root-associated microbiota as well as seedling vigor.
