# Supplementary material for: Soil domestication by rice cultivation results in plant-soil feedback through shifts in soil microbiota
Source: Genome Biol. 2019 Oct 24;20:221. doi: 10.1186/s13059-019-1825-x (PMC6814045; doi:10.1186/s13059-019-1825-x)
Supplement: Supplementary file 3 — Additional file 3. Review history. [file 13059_2019_1825_MOESM3_ESM.docx]

**Review history**

**First round of review**

**Reviewer 1**

Edwards et al investigated the impact of rice long-term cultivation on soil microbial communities and how these communities affect plant performance (plant soil feedback)

The main question; that is, what is the effect of long-term cultivation on plant soil feedback, is in my opinion not sufficiently substantiated. Although the authors give a nice overview of the differences between domesticated and non-domesticated soils, they do not answer or discuss sufficiently how long term cultivation negatively affects plant performance. Hence, this should be discussed in more detail.

Other comments:

Results

L117. You stated "consistent with our previous results". Which results?

L124-127. I think this refers to the analysis of multivariate homogeneity of group dispersions. Did you use betadisper function in vegan? Include this in your materials and methods section.

L 167-168. You mention that you experimental design tried to avoid confounding edaphic factors. Why didn't you take the same approach in your "soil domestication study". Did you domesticated vs non-domesticated soils have the same/similar physicochemical conditions?

I like that you start the results with a description of the experimental set-up, but I feel that sometimes this part is too long (e.g., L173-180).

L213 and others. It sound very weird when you refer to "The core rice depleted microbes". You should talk about the core microbiome of the native plants.

Discussion

The rhizosphere effect of rice has been demonstrated by the same authors. So that rice "domesticates" soil microbial communities makes sense. Especially considering that under continuous cultivation there is no real bulk soil, as this could have been rhizosphere soil at some point.

L366. Geographically identical areas do not guarantee that your domesticated/non-domesticated soils have similar physicochemical conditions.

L370-372. You mention that native plants do not seem to domesticate the soil microbial communities. Where the native plants present during the same period of time that rice? Maybe this is because native plants did not have the time to domesticate them.

L404-405. You mention that "the growth inhibition observed in our study does not appear to arise from specific microbial taxa". How about fungi, some plants (e.g., peanut) seems to accumulate fungal pathogens.

You should mention previous work reporting that rice seems to affect methanotrophs and methanogens (e.g., Lee et al (2015). Front Microbiol 6:639).

Methods

How many samples per experiment were sequenced?

What is UC mix?

Provide the reference for NINJA-OPS (L563).

You should mention that you use Bray-Curtis distances. Did you obtain the same results with other dissimilarity indexes?

According to a recent study (McKnight et al., https://doi.org/10.1111/2041-210X.13115) normalizing via proportions may be superior to other commonly used (included DESeq-VS) methods for comparing ecological communities.

**Reviewer 2**

Edwards et al proposes a study aimed at assessing if and how the soil microbiome is impacted by continuous cultivation of the same crop and if and how the microbiome of the "domesticated soil" by the crop affects plant growth. Starting from soils of fields with different cultivation regimes over time/vegetation management, it is proposed that the vegetation chronology/management has an effect in the selection and diversity of the soil prokaryote communities associated to rice used as the model crop for testing the effect on/exerted by the soil prokaryote microbiome. Prokaryote taxa associated to the differences between the treatments have been identified as domesticated by previous history of crop cultivation in the original soil. In a second part of the study, after extraction of the microbiomes from the soils, they are amended to a sterile inert substrate and their effects on rice growth is recorded. Similarly, the original soils are mixed with a standard plant growth matrix and the effect of the domesticated vs the non-domesticated soils is tested on the vigor of rice growth. Results indicated that the rice growth is linked to soil cultivation history and the soils and microbiomes from soils previously cultivated with rice reduce seedlings vigor. It is concluded that rice monoculture domesticates the soil microbiomes and selects microorganisms that favor plant vigor loss and potentially increase greenhouse gas emissions.

The topic of this study is of great and actual interest because it attempts to address the reasons that limits crop yields and connect such losses of growth yield to the soil microbiomes an aspect that is emerging as very important to maintain yields over time.

However, while several experiments are original and provide results of potential interest, such as the manipulative experiments of the soils and of the extracted microbiomes used for soil and microbiome transplants, I am strongly concerned by the premises of the experimental design that do not allow to get unambiguous conclusions and by misinterpretation of the results in the light of the experimenta conditions:

1)	The main conclusion of the first part of the study is that the cultivation history is the factor that determines soil domestication, i.e. a domestication of the microbiota that is different from that of the control soils that have not been cropped. However, the data fails to present the potential effect of the soil per se. How does any eventual difference in the soil chemistry and physics affect the microbiome structure? What is the weight of this factor respect to the plant cover? The study does not present any reference to the soil composition and structure that are known to be major factor implicated in the selection of the microbial communities.

2)	What is the effect of the agricultural practices? Are they stronger that those of the rice plants? Rice is cultivated under flooded conditions that generate anoxia, a factor dramatically affecting soil microbial diversity. The control soils are characterized by multiple variable factors respect to the rice monoculture: i) the type of vegetation cover, a lack of flooding conditions and probable differences in the intrinsic chemical-physical characteristics. How can be the different experiments compared and the effects of these factors disentangled?

3)	A message of the study is that rice monoculture selects for methanogenic archaea in soil and this represents a threat for the environment, because of the release of greenhouse gasses. After reading the manuscript I got the message that rice selects these archaea, while I guess the main factor is the cultivation under flooded conditions.

4)	The above factors (soil chemical-physical characteristics of the soils, water flooding management, other agricultural practices) represent major obvious differences among the different soils compared in the study and it is surprising that details on such important parameters are briefly mentioned without detailed explanations.

Below, are reported some specific comments that may help in improving the quality of the manuscript.

1.	Line 49: "Domestication of soil for agriculture". While this is an interesting concept that goes beyond the known commonly considered concept of "domestication" including "plant domestication", it should be placed in a context. It should require a formal conceptual definition, if not in the abstract, in the manuscript introduction.

2.	Lines 51-53: This sentence poses interesting conceptual questions: 1) How soil can be domesticated without a continuous cultivation? 2) Are there alternative ways other than continuous cultivation? 3) What is the meaning here with "continuous cultivation", continuity over time (of different crops or plants) or "monoculture"? I guess that a clear meaning is elusive because a careful contextualization and formal definition of "soil domestication" is missing. According to the rest of the manuscript the study is referring to rice continuous monoculture, so it is suggested to revise the text for terminology. In this case "rice monoculture" appears more appropriate than "rice long-term cultivation" in line 56.

3.	Line 67: "are likely determined". At lines 61-63 above, it is stated that "rice plants themselves, in addition to cultivation practices, are important drivers of the domestication process", while this term suggests that the plant only is responsible. To be consistent with the previous text, the sentence should be revised to indicate that the soil domestication is contributed by multiple factors.

4.	Lines 68-69: This sentence together with the previous is suggestive that rice plants determine the enrichment of methanogenic archaea (the "greenhouse gas emitting microbes"), while the agricultural practice of soil submersion with water (from the Materials and Methods of this study it is not fully clear weather the rice was cultivated under submersion or not. Description of procedures could be improved) is more important or at least contribute to their enrichment. Moreover, this sentence suggests that the change in the soil microbiota is responsible of the decreased plant performance, while this could be also determined by plant-driven allelopathy.

5.	Abstract: it is suggested to fully revise the abstract by using more appropriate terminology and avoiding arbitrary interpretation of the findings.

6.	Line 86: "Much…. Settings,". This is not correct. Similar findings to citation [8] have been observed in crop-cultivated soils as well after manipulative experiments on plant monoculture opposed to succession of different crops (Li et al. 2019 Legacy of land use history determines reprogramming of plant physiology by soil microbiome. The ISME Journal 13:738-751.). Please revise the text and include the citation indicated.

7.	Lines 86-88: Please eliminate the part of the sentence referring to climate change. The process of soil domestication is not directly related to climate change and is not investigated because of the climate change.

8.	Line 88: As indicated above, it would be necessary to clarify the terminology. Here is a place in the introduction where such a formal conceptual definition of "soil domestication" could be included. Similarly, it is suggested to clarify the concept of monoculture vs long term cultivation etc.

9.	Line 112: It should be better detailed here the use history of these soils. Were they used for rice as a monoculture for long time or were they used for rice but under crop rotation?

10.	Line 114: Similarly to the previous comment, the plant cover features of the two uncultivated soil should be explained. Were they plant-free soils? In case they were colonized by plants what kind of plants were growing in these soils? What was the floristic composition?

11.	Line 116: "microbiota". Do you mean "microbiota taxonomic composition"?

12.	Line 116: "microbiota", which microbiota? The whole microbiota or bacteria, archaea, …? Please specify. The same for "16S rRNA gene", was it of bacteria, archaea or both? Please, check all the manuscript for the appropriate use of terms.

13.	Line 118: "microbial communities" reads "bacterial (or prokaryote) communities". Please, check throughout the manuscript the correct use of the term.

14.	Line 118-120: This sentence suggests the reader that the measured difference in the prokaryote communities is associated to the fact that two soils were cultivated with rice while the other two were uncultivated soils. But the differences could be explained by a different soil geochemistry or by the different original vegetation cover, for instance. What was the difference in the geochemical and physical properties of the four soil types? Can this be excluded as a discrimination factor? The differences in the prokaryote community could be explained by the differences in the original vegetation type cover rather than the fact that one soil group was cultivated and the other was not? To exclude that factors other than soil cultivation history, such as soil geochemical/physical nature, are involved in the community segregation, an ordination of the soil based on soil geochemical/physical data should be presented. If a data ordination similar to that obtained with the prokaryote microbiome data occur, what is the factor explaining the observed differences? The soil use history or the soil geochemistry?

15.	Figure 1: the figure presentation is inaccurate. "Bray" reads "Bray-Curtis". The x-axis of the ordination plot lacks the PCo1 component contribution.

16.	Line 122: "were significantly affected by soil history", please, see comment n. 14.

17.	Line 131-133: This sentence should be revised because it is not clear what "in a state more similar to the rice rhizosphere" means. 'State' of what? I guess the Authors intend to say that respect to the uncultivated soil, the bulk soil of rice fields has a microbial community enriched of prokaryotes capable to access the rice rhizosphere.

18.	Line 168: In these reconstructed experiments the potential confounding effect of the variable soil chemico-physical properties is considered and managed in the design of the experiments (so, it is not clear why this was not done before). However, it should be commented if and how the microbiome extracted from the different treatments is affected by the nature (chemico-physical properties) of the original soils.

19.	Lines 225-273: It is not fully clear why these analyses were performed. To demonstrate that the root of different plants hosts different microbiomes and that there is compartment (rhizosphere vs endosphere vs bulk soil) effect? Plant species and compartment effects on root-associated microbiomes are very well known with hundreds of published studies. It is not clear how this section may contribute to the purpose and the novelty of the study.

20.	Lines 253-255: "…finding that the endosphere microbiome was slightly more affected by host species…than the endosphere microbiome…"? I guess this sentence intends to compare endosphere with rhizosphere.

21.	Lines 276-286: Please, see comment 19. The usefulness of this section is not clear

22.	Line293-296: These include several potential obligate anaerobes. Are these selected by rice or are they selected by the flooding conditions that generate anaerobic niches?

23.	Lines 301-309: Please, refer to comment 22.

24.	Lines 310-331: These refer to the aerobic methanotrophs that are related to the presence of methane. Please refer again to comment 22.

25.	Lines 333-358 Please refer to comment 22.

26.	Discussion: I suggest to revise the discussion in the light of the other comments.

27.	Lines 463-526: This is a study on soil use history on the soil microbiomes and the reciprocal effect of the selected soils on plant growth. Microorganisms from soil under long-term monoculture of rice and from non-cultivated soils with spontaneous vegetation are the origin and the subject of the study. For the nature of the study and a correct understanding and assessment of the results, detailed quantitative descriptions of 1) the original soils (including their chemical-physical characteristics), 2) their floristic cover (for non-cultivated soils) and 3) the adopted agricultural practices, are indispensable. For instance, methanogenic archaea have been detected in different experiments with the cultivation of rice and it is concluded that rice selects for these anaerobic archaea. But this is expected if the soils are under flooded cultivation conditions. So, a question is if the methanogens are selected by the rice plant itself or by the flooding practice. Please, provide such information to remove ambiguities on what cause what. What one can deduct from the actual text (lines 477, 492, 502, 511-512) is that soils are inundated with water for periods of ten or more days or kept under bottom irrigation. However, this does not clarify the level of water saturation of the soil that would make a big difference for the selection of anaerobic prokaryotes like methanogens and others found in the study.

**Response to reviewers**

Reviewer 1

Edwards et al investigated the impact of rice long-term cultivation on soil microbial

communities and how these communities affect plant performance (plant soil

feedback)

The main question; that is, what is the effect of long-term cultivation on plant soil

feedback, is in my opinion not sufficiently substantiated. Although the authors give a

nice overview of the differences between domesticated and non-domesticated soils,

they do not answer or discuss sufficiently how long term cultivation negatively affects

plant performance. Hence, this should be discussed in more detail.

*We thank the reviewer for bringing to our attention that the main question was not*

*framed with sufficient clarity to show how this study had addressed it.*

*In this revision, we have tried to make how we addressed the central question (i.e.*

*effect of long-term cultivation on plant-soil feedback) clearer by splitting the question*

*into three separate components:*

*1. Does long term rice cultivation change the bacterial and archaeal components of*

*the soil microbiome?*

*2. If so, is the rice plant itself a driver of the soil domestication process at the microbial*

*level?*

*3. What is the impact on host plant vigor of domesticated microbiomes compared to*

*microbiomes of undomesticated soils: Growth promotion (e.g. due to selection of*

*favorable microbiota by the host plant) vs. No difference or Growth inhibition?*

*We have now specified these objectives of the study in the Introduction (L. 106 - 113),*

*and the Results section of the manuscript has been re-organized to address these*

*individual questions more clearly.*

Other comments:

Results

L117. You stated "consistent with our previous results". Which results?

We have now included citations to our previous work (Edwards et al. 2015; Santos-

Medellín et al. 2017; and Edwards, et al. 2018) to support this claim (L. 128).

L124-127. I think this refers to the analysis of multivariate homogeneity of group

dispersions. Did you use betadisper function in vegan? Include this in your materials

and methods section.

*Yes, we did use the betadisper function to calculate distances to group centroids. The*

*previous Materials and Methods included a citation to using the betadisper function,*

*but lacked a thorough explanation for why this function was used. We have now*

*amended this particular section in the Materials and Methods to better reflect how this*

*function was used (L. 589).*

L 167-168. You mention that you experimental design tried to avoid confounding

edaphic factors. Why didn't you take the same approach in your "soil domestication

study". Did you domesticated vs non-domesticated soils have the same/similar

physicochemical conditions?

*We have now conducted a soil chemistry analysis, finding that soil chemistry*

*differences appear to correlate with geography rather than by soil history (L 123 – 125,*

*Suppl. Fig. 2 and Suppl. Table 1)*

I like that you start the results with a description of the experimental set-up, but I feel

that sometimes this part is too long (e.g., L173-180).

*We agree with the reviewer, and have now shortened this section.*

L213 and others. It sound very weird when you refer to "The core rice depleted

microbes". You should talk about the core microbiome of the native plants.

*We agree with the reviewer that the term “core rice depleted microbes” is an*

*unnecessarily confusing term. We now refer to these specific microbes as “native plant*

*core enriched microbes”, as the reviewer suggested.*

Discussion

The rhizosphere effect of rice has been demonstrated by the same authors. So that

rice "domesticates" soil microbial communities makes sense. Especially considering

that under continuous cultivation there is no real bulk soil, as this could have been

rhizosphere soil at some point.

L366. Geographically identical areas do not guarantee that your domesticated/nondomesticated

soils have similar physicochemical conditions.

*We agree with this point. However, we used the term “geographically identical areas”*

*only to indicate that the non-cultivated soils are likely representative for the*

*uncultivated state of a given rice field in the same environment. We have now added a*

*statement to this sentence to make this point clearer (L. 362).*

L370-372. You mention that native plants do not seem to domesticate the soil microbial

communities. Where the native plants present during the same period of time that rice?

Maybe this is because native plants did not have the time to domesticate them.

*The question that the reviewer brings up is interesting: could native plants not be*

*domesticating soil microbiota because they do not interact with soil microbiota long*

*enough to have a detectable effect? We based our assertion that the native plants do*

*not have a demonstrable role in domesticating rice field soils because 1) unplanted*

*soils (“bulk soil”) from cultivated fields have microbiomes more similar to rice*

*rhizosphere microbiomes, and 2) the native plants host distinct microbiomes from each*

*other and from the bulk soil. Regarding the specific comment of the reviewer, the*

*native plants we surveyed are common weeds, which typically have shorter lifespans*

*than the rice plants. In addition, native plants (i.e. weeds) are controlled by farmers and*

*therefore do not comprise an appreciable amount of the total plants in a rice field.*

*While we did not test whether native plants can significantly shift the soil microbiota on*

*a field level, there are multiple reports that soil microbial communities are influenced by*

*the native plant cover at a specific site (Prober et al. 2015; Schlatter et al. 2015).*

*Therefore, we have edited our comment to better reflect the possibility that native*

*plants may play a stronger role in rice field soil domestication if weed management*

*were to differ (L 368 - 369).*

L404-405. You mention that "the growth inhibition observed in our study does not

appear to arise from specific microbial taxa". How about fungi, some plants (e.g.,

peanut) seems to accumulate fungal pathogens.

*We could not find the specific publication the reviewer is referring to, but we did find a*

*paper by Li et al, 2019 on effects of mono-cropping vs. crop-rotation in peanut*

*cultivation, which suggests that bacteria, but not fungi, are responsible for deleterious*

*growth when grown in monoculture soils. They come to this conclusion because they*

*found genes related to responses to bacterial pathogens upregulated when in*

*monoculture conditions, but no upregulation of genes involved in fungal pathogen*

*response.*

*“Furthermore, many genes involved in plant responses to bacterial factors, including*

*flagellin and EF-Tu, were upregulated in the monocropped peanut (Supplementary Fig.*

*S5). However, the expression of most genes involved in responses to fungal*

*pathogens remained apparently unchanged.”*

*We do agree however that the sentence highlighted by the reviewer is too broad for the*

*scope of this study, and we have now revised the sentence to say “specific prokaryotic*

*taxa” instead of “specific microbial taxa” (L. 406 - 407). We have further included a*

*statement in the manuscript that because we did not survey fungal communities in the*

*microbiome, we cannot exclude that specific fungal taxa might contribute to adverse*

*plant performance in domesticated soils (L. 419 - 424).*

You should mention previous work reporting that rice seems to affect methanotrophs

and methanogens (e.g., Lee et al (2015). Front Microbiol 6:639).

*We have now added this citation, as well as others describing methanogen /*

*methanotroph patterns in rice field soils (L 244).*

Methods

How many samples per experiment were sequenced?

*We have added a new supplementary figure (Suppl. Fig. 1) describing the design for*

*each experiment performed for this study, as well as the number of replicates*

*sequenced for each treatment / sample type combination.*

What is UC mix?

*We have revised the Methods section as well as the Results section to better describe*

*the composition of UC Mix. We have also inserted a link to the webpage which lists the*

*recipe (L. 5235 - 527).*

Provide the reference for NINJA-OPS (L563).

*We have now added the proper citation for the NINJA-OPS software.*

You should mention that you use Bray-Curtis distances. Did you obtain the same

results with other dissimilarity indexes?

*We have now re-analyzed the data using weighted and unweighted Unifrac as*

*alternative dissimilarity metrics, and we find that the results are essentially the same (L.*

*133 and L. 197). The results using these metrics are presented as Supplementary*

*Figures 3 and 6 in the revised manuscript. Permanova statistics were also calculated*

*and included as Supplementary Tables 3 and 8.*

According to a recent study (McKnight et al., https://doi.org/10.1111/2041-210X.13115)

normalizing via proportions may be superior to other commonly used (included DESeq-

VS) methods for comparing ecological communities.

*We thank the reviewer for the reference. However, there is currently no consensus in*

*the field concerning an optimal method for normalization. We prefer to use variance*

*stabilizing transformations when working with our data. There have been numerous*

*studies advocating for this technique (Nguyen and Holmes 2019; McMurdie and*

*Holmes 2014) - and we agree with the authors. 16S rRNA sequencing data, much like*

*RNA-seq data, exhibits a relationship between means and variances of OTUs, i.e.*

*OTUs with higher relative abundance in the dataset also have higher variances. If left*

*uncorrected, community wide statistics are then biased towards the highly abundant*

*taxa when using weighted dissimilarity metrics. Multiple studies advocate that rare / low*

*abundant taxa may be important regulators for microbiome assembly and ecosystem*

*processes, therefore we do not want to negate their contribution (Jousset et al. 2017).*

Reviewer #2: Edwards et al proposes a study aimed at assessing if and how the soil

microbiome is impacted by continuous cultivation of the same crop and if and how the

microbiome of the "domesticated soil" by the crop affects plant growth. Starting from

soils of fields with different cultivation regimes over time/vegetation management, it is

proposed that the vegetation chronology/management has an effect in the selection

and diversity of the soil prokaryote communities associated to rice used as the model

crop for testing the effect on/exerted by the soil prokaryote microbiome. Prokaryote

taxa associated to the differences between the treatments have been identified as

domesticated by previous history of crop cultivation in the original soil. In a second part

of the study, after extraction of the microbiomes from the soils, they are amended to a

sterile inert substrate and their effects on rice growth is recorded. Similarly, the original

soils are mixed with a standard plant growth matrix and the effect of the domesticated

vs the non-domesticated soils is tested on the vigor of rice growth. Results indicated

that the rice growth is linked to soil cultivation history and the soils and microbiomes

from soils previously cultivated with rice reduce seedlings vigor. It is concluded that rice

monoculture domesticates the soil microbiomes and selects microorganisms that favor

plant vigor loss and potentially increase greenhouse gas emissions.

The topic of this study is of great and actual interest because it attempts to address the

reasons that limits crop yields and connect such losses of growth yield to the soil

microbiomes an aspect that is emerging as very important to maintain yields over time.

However, while several experiments are original and provide results of potential

interest, such as the manipulative experiments of the soils and of the extracted

microbiomes used for soil and microbiome transplants, I am strongly concerned by the

premises of the experimental design that do not allow to get unambiguous conclusions

and by misinterpretation of the results in the light of the experimenta conditions:

1)The main conclusion of the first part of the study is that the cultivation history is the

factor that determines soil domestication, i.e. a domestication of the microbiota that is

different from that of the control soils that have not been cropped. However, the data

fails to present the potential effect of the soil per se. How does any eventual difference

in the soil chemistry and physics affect the microbiome structure? What is the weight of

this factor respect to the plant cover? The study does not present any reference to the

soil composition and structure that are known to be major factor implicated in the

selection of the microbial communities.

*We agree with the reviewer that inclusion of the soil nutrient profiles into the*

*manuscript is important for defining the nutritional state of each soil and to potentially*

*explain the differences that we observe between domesticated and uncultivated soils.*

*We have now performed the soil nutrient analysis for the soils, and provided these data*

*in Supplementary Table 1. We find that soils nutrient profiles cluster more by*

*geographic location than by soil history (L. 123 - 125). We performed a principal*

*coordinate analysis on soil NO3, P, K, Ca, Mg, Na, organic matter, cation exchange*

*capacity and pH and have now included this information in Supplementary Figure 2B-C*

*.*

*We have also surveyed the plant diversity growing in each uncultivated site, and find*

*that each site was host to differing plant communities, with very little overlap (shown in*

*Supplementary Figure 2D). The details have been added to the manuscript (L. 125 -*

*127), and a further explanation can be found in response to Specific Comment #10.*

2)What is the effect of the agricultural practices? Are they stronger that those of the

rice plants? Rice is cultivated under flooded conditions that generate anoxia, a factor

dramatically affecting soil microbial diversity. The control soils are characterized by

multiple variable factors respect to the rice monoculture: i) the type of vegetation cover,

a lack of flooding conditions and probable differences in the intrinsic chemical-physical

characteristics. How can be the different experiments compared and the effects of

these factors disentangled?

*It was not our goal to disentangle these factors at the outset when designing these*

*experiments. Our study was designed to investigate the degree to which the*

*culmination of these agricultural practices influence rice microbiome assemblages in*

*comparison to soil which is not used for growing crops. The next step was to ask*

*whether the rice plants themselves play a part in shifting the soil microbiota towards a*

*domesticated state. We agree with the reviewer that the question of how each*

*agricultural practice unique to rice influences the domestication of soil microbiota would*

*be a fascinating aspect to study - but our experimental designs were not equipped for*

*these questions. We feel that this does not detract from the message of our study,*

*which is that long term monoculture of rice shifts microbial assemblages towards a*

*community composition distinct from uncultivated soils. We do show that the different*

*cultivated soils behave similarly with respect to their compositional changes and*

*phenotypic effects on the plants, suggesting that the variables indicated by the*

*reviewer are secondary in their effects.*

3)A message of the study is that rice monoculture selects for methanogenic archaea in

soil and this represents a threat for the environment, because of the release of

greenhouse gasses. After reading the manuscript I got the message that rice selects

these archaea, while I guess the main factor is the cultivation under flooded conditions.

*We are proposing a synergistic effect of the two mechanisms. Our results suggest that*

*under flooded conditions rice is either enriching or allowing residence of methanogenic*

*archaea both in the rhizosphere and root while the native plants growing under flooded*

*conditions either eliminate or are not colonized by methanogens to the same degree as*

*rice. There appears to be a two-step process: flooding of soils promotes favorable*

*conditions for growth of methanogens and then rice roots provide favorable*

*colonization conditions for the methanogens, while native plants are unfavorable. We*

*agree with the reviewer that this point was not discussed adequately and have now*

*expanded the discussion to make it clearer (L. 442 - 447). We thank the reviewer for*

*helping to add detail to this model which we have now added to the discussion in the*

*manuscript.*

4)The above factors (soil chemical-physical characteristics of the soils, water flooding

management, other agricultural practices) represent major obvious differences among

the different soils compared in the study and it is surprising that details on such

important parameters are briefly mentioned without detailed explanations.

*The main focus of the manuscript and its conclusions are not about differences*

*between field soils, but about changes to the soil microbiota that appear universal to*

*cultivation despite field/management differences, and their consequences to plant*

*growth. Thus, we are not attempting to attribute specific changes to particular aspects*

*of the soils from different fields, but rather to identify emergent properties that are in*

*common to the cultivated soils, and distinct from the uncultivated soils. To*

*demonstrate that soil microbiota rather than soil physical-chemical properties are*

*responsible for the observed phenotypic effects of domesticated soils, the greenhouse*

*assays were performed under conditions where the same sterilized potting medium*

*was used for all plants. The soil suspensions from different sources were inoculated at*

*dilution factors of 0.5%, which would be a negligible contribution to the potting medium,*

*but were sufficient for establishment of a representative microbiome within the timeframe*

*of the experiments. We do agree with the reviewer about the need for detailed*

*characterization of the cultivated and uncultivated soils by chemical analyses, and*

*these have now been provided (please see response to comment #1 above).*

Below, are reported some specific comments that may help in improving the quality of

the manuscript.

1.Line 49: "Domestication of soil for agriculture". While this is an interesting concept

that goes beyond the known commonly considered concept of "domestication"

including "plant domestication", it should be placed in a context. It should require a

formal conceptual definition, if not in the abstract, in the manuscript introduction.

*We agree with the reviewer and have now defined soil domestication in the abstract*

*and the introduction (L. 50-51 and 87 - 88).*

2.Lines 51-53: This sentence poses interesting conceptual questions: 1) How soil can

be domesticated without a continuous cultivation? 2) Are there alternative ways other

than continuous cultivation? 3) What is the meaning here with "continuous cultivation",

continuity over time (of different crops or plants) or "monoculture"? I guess that a clear

meaning is elusive because a careful contextualization and formal definition of "soil

domestication" is missing. According to the rest of the manuscript the study is referring

to rice continuous monoculture, so it is suggested to revise the text for terminology. In

this case "rice monoculture" appears more appropriate than "rice long-term cultivation"

in line 56.

*Questions 1 and 2 are fascinating inquiries that we hope to follow up on in future*

*studies. We have posed these questions in our Conclusion section as outstanding*

*Powered by Editorial Manager® and ProduXion Manager® from Aries Systems Corporation*

*questions (L. 465 - 468). We have now defined soil domestication in the abstract and*

*the introduction. We agree with the point about monoculture, and have now revised the*

*text in the manuscript to read “continuous rice monoculture” instead of “long term rice*

*Cultivation.”*

3.Line 67: "are likely determined". At lines 61-63 above, it is stated that "rice plants

themselves, in addition to cultivation practices, are important drivers of the

domestication process", while this term suggests that the plant only is responsible. To

be consistent with the previous text, the sentence should be revised to indicate that the

soil domestication is contributed by multiple factors.

*We have edited this sentence to reflect that the rice plants are at least partially*

*responsible for the observed bacterial and archaeal community shifts in domesticated*

*soils (L. 61, 70). We found a similar instance in the Conclusion section that has now*

*also been edited (L. 462).*

4.Lines 68-69: This sentence together with the previous is suggestive that rice plants

determine the enrichment of methanogenic archaea (the "greenhouse gas emitting

microbes"), while the agricultural practice of soil submersion with water (from the

Materials and Methods of this study it is not fully clear weather the rice was cultivated

under submersion or not. Description of procedures could be improved) is more

important or at least contribute to their enrichment. Moreover, this sentence suggests

that the change in the soil microbiota is responsible of the decreased plant

performance, while this could be also determined by plant-driven allelopathy.

*We have amended the Materials and Methods under the Soil Domestication Study*

*header to better reflect the manner in which the rice plants were grown. Both*

*domesticated and undomesticated soils were placed under flooded conditions for the*

*experiment in our greenhouse.*

*In our seedling vigor experiments, the experimental design makes it likely that the*

*variation we see in plant performance is due to plant-microbe interactions. A sterilized*

*common growth substrate (either calcined clay or a potting soil mix) was inoculated*

*with 1% v/v field soil:growth substrate or 200 fold dilutions of soil suspension.*

*Allelopathy could not explain the results because the concentration of plant metabolites*

*entering the soil would be far below the necessary concentration for allelopathy. Rice*

*plants typically exhibit limited autotoxicity from allelopathy, requiring in most cases >*

*100 mg / L of rice root exudate to yield an observable effect (Ma et al. 2014) - a*

*concentration we would not be able to achieve using our inoculation strategy. We have*

*now added this reasoning to the discussion section (L. 383 - 386).*

5.Abstract: it is suggested to fully revise the abstract by using more appropriate

terminology and avoiding arbitrary interpretation of the findings.

*The Abstract has been revised as suggested.*

6.Line 86: "Much…. Settings,". This is not correct. Similar findings to citation [8] have

been observed in crop-cultivated soils as well after manipulative experiments on plant

monoculture opposed to succession of different crops (Li et al. 2019 Legacy of land

use history determines reprogramming of plant physiology by soil microbiome. The

ISME Journal 13:738-751.). Please revise the text and include the citation indicated.

*We thank the reviewer for bringing this recent insightful paper to our notice, and have*

*now added the Li et al. 2019 citation to our Introduction and Discussion sections to*

*give further context to our manuscript. We have amended the sentence highlighted by*

*this comment to now read “Less is known about plant-soil feedback in agricultural*

*settings about plant-soil feedback in agricultural settings...” (L. 87) We feel that this*

*better reflects the state of the literature - plant-soil feedbacks have mainly been*

*studied in natural ecosystems, but have recently emerged as a focal point in*

*agricultural systems. We also agree that the Li et al. study examines monoculture vs.*

*crop-rotation in peanuts, and not the effects of soil domestication vs. uncultivated soils.*

*Because that study was based on a single field in which all rotation plots had been*

*planted with potato the previous year, the generality of their conclusions is somewhat*

*restricted.*

7.Lines 86-88: Please eliminate the part of the sentence referring to climate change.

The process of soil domestication is not directly related to climate change and is not

investigated because of the climate change.

*We have now removed this part of the sentence, replacing it with a definition of soil*

*Domestication.*

8.Line 88: As indicated above, it would be necessary to clarify the terminology. Here is

a place in the introduction where such a formal conceptual definition of "soil

domestication" could be included. Similarly, it is suggested to clarify the concept of

monoculture vs long term cultivation etc.

*See response to Comment 7. In addition, we have now replaced all mentions of “longterm*

*cultivation” with “continuous monoculture cultivation” to more accurately describe*

*the conditions of our experiments.*

9.Line 112: It should be better detailed here the use history of these soils. Were they

used for rice as a monoculture for long time or were they used for rice but under crop

Rotation?

*We agree with the reviewer that cultivation details for each site are important to*

*describe. We have therefore included the details that each domesticated site had*

*cultivated rice in monoculture for at least 8 growing seasons (L. 121 - 122).*

10.Line 114: Similarly to the previous comment, the plant cover features of the two

uncultivated soil should be explained. Were they plant-free soils? In case they were

colonized by plants what kind of plants were growing in these soils? What was the

floristic composition?

*We have now added a figure (Suppl. Fig. 2D) describing the floristic composition at*

*each non-cultivated site. We found minimal overlap between the three uncultivated*

*sites. We thank the reviewer for this suggestion – these observations provide a*

*potential explanation for our finding that bacterial and archaeal communities*

*assembled from uncultivated soils show greater between-soil variation than those*

*assembled from rice domesticated soils. We have added this explanation in the*

*Results section in lines 140 - 143.*

11.Line 116: "microbiota". Do you mean "microbiota taxonomic composition"?

*The reviewer is correct- we have fixed this.*

12.Line 116: "microbiota", which microbiota? The whole microbiota or bacteria,

archaea, …? Please specify. The same for "16S rRNA gene", was it of bacteria,

archaea or both? Please, check all the manuscript for the appropriate use of terms.

*Instead of editing the manuscript to read “bacterial and archaeal microbiota” or*

*“prokaryotic microbiota” for every instance of the word “microbiota” which would be*

*unwieldy, we have included a sentence at the end of the introduction to clarify that only*

*the bacterial and archaeal taxonomic component are being surveyed (L 111). We feel*

*this is a fair practice: if there are variations in the bacterial or archaeal communities it*

*follows that microbiota taken as a whole have variations.*

13.Line 118: "microbial communities" reads "bacterial (or prokaryote) communities".

Please, check throughout the manuscript the correct use of the term.

*Please see response to Comment 12.*

14.Line 118-120: This sentence suggests the reader that the measured difference in

the prokaryote communities is associated to the fact that two soils were cultivated with

rice while the other two were uncultivated soils. But the differences could be explained

by a different soil geochemistry or by the different original vegetation cover, for

instance. What was the difference in the geochemical and physical properties of the

four soil types? Can this be excluded as a discrimination factor? The differences in the

prokaryote community could be explained by the differences in the original vegetation

type cover rather than the fact that one soil group was cultivated and the other was

Powered by Editorial Manager® and ProduXion Manager® from Aries Systems Corporation

not? To exclude that factors other than soil cultivation history, such as soil

geochemical/physical nature, are involved in the community segregation, an ordination

of the soil based on soil geochemical/physical data should be presented. If a data

ordination similar to that obtained with the prokaryote microbiome data occur, what is

the factor explaining the observed differences? The soil use history or the soil

Geochemistry?

*We have now conducted a soil chemistry analysis, finding that soil nutrient profiles*

*cluster more by geography than soil history. For example Arbuckle cultivated and non*

*cultivated cluster together, Biggs cultivated and uncultivated soils cluster together, and*

*Sacramento (non cultivated) is closest to Davis soil (a fallow field). We have added*

*these results as Supplemental figure 2A-C.*

*We have now also surveyed the floristic composition at each site, finding that each*

*uncultivated site contains differing plant communities with very little overlap (Suppl. Fig.*

*2D). Only the Davis soil is devoid of plants - the soil is plowed every year but not*

*cultivated or flooded.*

15.Figure 1: the figure presentation is inaccurate. "Bray" reads "Bray-Curtis". The xaxis

of the ordination plot lacks the PCo1 component contribution.

*We thank the reviewer for noticing this omission. We have now added the axis label to*

*the ordination in Figure 1.*

16.Line 122: "were significantly affected by soil history", please, see comment n. 14.

*Please see response to comment 14. Given these two findings, we feel that our*

*statement that plant associated microbiota are affected by soil cultivation history is now*

*supported vs. the alternate possibilities suggested in comment #14.*

17.Line 131-133: This sentence should be revised because it is not clear what "in a

state more similar to the rice rhizosphere" means. 'State' of what? I guess the Authors

intend to say that respect to the uncultivated soil, the bulk soil of rice fields has a

microbial community enriched of prokaryotes capable to access the rice rhizosphere.

*The rationale behind this analysis was to compare the magnitude of the compositional*

*shift between soil and rhizosphere communities across cultivation histories. We*

*observed that, relative to uncultivated samples, domesticated rhizospheres are*

*compositionally more similar to their respective bulk soils. Given that rhizosphere*

*acquisition is a host-mediated process, this result sets the stage for a potential role of*

*rice roots in the compositional differences observed between cultivated and*

*uncultivated soils. We have rewritten this section to better convey this message (L. 144*

*- 152).*

18.Line 168: In these reconstructed experiments the potential confounding effect of the

variable soil chemico-physical properties is considered and managed in the design of

the experiments (so, it is not clear why this was not done before). However, it should

be commented if and how the microbiome extracted from the different treatments is

affected by the nature (chemico-physical properties) of the original soils.

*We have now mentioned in the leadup to describing the setup of this experiment that*

*soil nutrient composition varies by geography (123 - 125).*

19.Lines 225-273: It is not fully clear why these analyses were performed. To

demonstrate that the root of different plants hosts different microbiomes and that there

is compartment (rhizosphere vs endosphere vs bulk soil) effect? Plant species and

compartment effects on root-associated microbiomes are very well known with

hundreds of published studies. It is not clear how this section may contribute to the

purpose and the novelty of the study.

*In light of this comment, we have thoroughly reorganized and reworded the Results*

*section of this manuscript. The changes include the ordering of the Results section and*

*eliminating some non-essential details (e.g., phylum level statistics between native*

*plants and rice microbiomes), to help clarify the narrative and rationale behind the*

*experiments.*

*As the reviewer pointed out elsewhere, it was not clear from the results of our first*

*experiment - where we compare prokaryotic microbiomes acquired in uncultivated and*

*rice monoculture soils- whether the rice plants themselves play a major role in the soil*

*domestication process, because transitions in soil microbiota could result from a variety*

*of cultivation practices (like flooding, tillage, nutrient addition, etc.). This experiment*

*and the results obtained are critical to elucidating the potential role played by rice*

*plants in shifting the soil microbiota for the following reasons:*

*1) They show that the rice plant rhizosphere supports bacterial and archaeal*

*microbiota much more similar to the bulk soil of the cultivated fields, than do the*

*rhizospheres of the native plants in the same field. As also shown in this study, rice*

*plants can still acquire a rhizosphere microbiome distinct from the bulk soil, if that soil*

*was previously uncultivated (i.e., rice is not deficient in the capacity to assemble a*

*distinct rhizosphere community from soil microbiota).*

*2) It allowed us to identify microbes that are specifically enriched in rice compared to*

*other plants that are all growing in a domesticated soil under flooding and other*

*managed conditions. We reasoned that if the rice plants are partially responsible for*

*soil domestication, then we should see a significant overlap between the microbes*

*enriched in the microbiomes of plants growing in domesticated soils (our first*

*experiment), and the core microbes that are enriched in the rice microbiome compared*

*to the native plants. We found that this is indeed the case (Fig 5).*

20.Lines 253-255: "…finding that the endosphere microbiome was slightly more

affected by host species…than the endosphere microbiome…"? I guess this sentence

intends to compare endosphere with rhizosphere.

*We thank the reviewer for bringing this error to our notice. We have now corrected the*

*sentence to read “...finding that the endosphere microbiome was slightly more affected*

*by host species (R2 = 0.42, P < 0.001, Sup .Table 7) than the rhizosphere microbiome*

*(R2 = 0.35, P < 0.001, Sup. Table 7).”*

21.Lines 276-286: Please, see comment 19. The usefulness of this section is not clear

*Please see our response to Comment 19.*

22.Line293-296: These include several potential obligate anaerobes. Are these

selected by rice or are they selected by the flooding conditions that generate anaerobic

Niches?

*Because the native plants were growing along with rice in the same flooded, anaerobic*

*environment, the data suggest that rice plants are selecting these microbes, or at least*

*not restricting their colonization, which differs from the native aquatic plants. We have*

*now edited the manuscript throughout to make it clear that all of the plants in this*

*experiment were growing under the same submerged conditions in a domesticated rice*

*field.*

23.Lines 301-309: Please, refer to comment 22.

*Please see response to Comment 22.*

24.Lines 310-331: These refer to the aerobic methanotrophs that are related to the

presence of methane.

*Please refer again to comment 22.*

25.Lines 333-358 Please refer to comment 22.

*Please see response to Comment 22*

26.Discussion: I suggest to revise the discussion in the light of the other comments.

*The Discussion has been revised as suggested.*

27.Lines 463-526: This is a study on soil use history on the soil microbiomes and the

reciprocal effect of the selected soils on plant growth. Microorganisms from soil under

long-term monoculture of rice and from non-cultivated soils with spontaneous

vegetation are the origin and the subject of the study. For the nature of the study and a

correct understanding and assessment of the results, detailed quantitative descriptions

of 1) the original soils (including their chemical-physical characteristics), 2) their floristic

cover (for non-cultivated soils) and 3) the adopted agricultural practices, are

indispensable. For instance, methanogenic archaea have been detected in different

experiments with the cultivation of rice and it is concluded that rice selects for these

anaerobic archaea. But this is expected if the soils are under flooded cultivation

conditions. So, a question is if the methanogens are selected by the rice plant itself or

by the flooding practice. Please, provide such information to remove ambiguities on

what cause what. What one can deduct from the actual text (lines 477, 492, 502, 511-

512) is that soils are inundated with water for periods of ten or more days or kept under

bottom irrigation. However, this does not clarify the level of water saturation of the soil

that would make a big difference for the selection of anaerobic prokaryotes like

methanogens and others found in the study.

*Please see responses to comments #14, #19 and #22. We agree with the reviewer that*

*the details of irrigation for this experiment were insufficient. We have clarified now in*

*the Results and Methods section that all experiments were conducted under*

*submerged conditions.*

**Second round of review**

**Reviewer 1**

I am happy with the new version of the manuscript.

There are some spelling/formatting mistakes, but I assume they will be corrected during the editing process.

**Reviewer 2**

Edwards et al made an excellent work in revising the manuscript that is now much improved and clear.

I have few minor comments:

1. Line 58: “We show here that continuous monoculture (>8 growing seasons) of the major food crop rice is…”. This should be changed with “We show here that continuous monoculture (>8 growing seasons) of the major food crop rice under flooding conditions is…”.

2. Line 120: I recommend to include Figure S1 as a full figure in the main manuscript. This is very important for understanding the manuscript. In the legend “16S” reads “16S rRNA gene”. In the legend should be also indicated the meaning of NA that appears to be different from panels A and B (not applicable) and panel C (?).

3. Line 145: “select”? Is it “selected”?

4. Line 361: Two different areas cannot be geographically identical, geographically close, but not identical.
